# Pharmacological Evidence of the Important Roles of CCR1 and CCR3 and Their Endogenous Ligands CCL2/7/8 in Hypersensitivity Based on a Murine Model of Neuropathic Pain

**DOI:** 10.3390/cells12010098

**Published:** 2022-12-26

**Authors:** Katarzyna Pawlik, Katarzyna Ciapała, Agata Ciechanowska, Klaudia Kwiatkowski, Joanna Mika

**Affiliations:** Department of Pain Pharmacology, Maj Institute of Pharmacology, Polish Academy of Sciences, 31-343 Krakow, Poland

**Keywords:** CCR1, CCR3, neuropathic pain, bindarit, chemokines, neuroinflammation, male, female

## Abstract

Neuropathic pain treatment remains a challenging issue because the therapies currently used in the clinic are not sufficiently effective. Moreover, the mechanism of neuropathy is still not entirely understood; however, much evidence indicates that chemokines are important factors in the initial and late phases of neuropathic pain. To date, the roles of CCR1, CCR3 and their endogenous ligands have not been extensively studied; therefore, they have become the subject of our research. In the present comprehensive behavioral and biochemical study, we detected significant time-dependent and long-lasting increases in the mRNA levels of CCR1 and/or CCR3 ligands, such as CCL2/3/4/5/6/7/8/9, in the murine spinal cord after chronic constriction injury of the sciatic nerve, and these increases were accompanied by changes in the levels of microglial/macrophage, astrocyte and neutrophil cell markers. ELISA results suggested that endogenous ligands of CCR1 and CCR3 are involved in the development (CCL2/3/5/7/8/9) and persistence (CCL2/7/8) of neuropathic pain. Moreover, intrathecal injection of CCL2/3/5/7/8/9 confirmed their possible strong influence on mechanical and thermal hypersensitivity development. Importantly, inhibition of CCL2/7/8 production and CCR1 and CCR3 blockade by selective/dual antagonists effectively reduced neuropathic pain-like behavior. The obtained data suggest that CCL2/7/8/CCR1 and CCL7/8/CCR3 signaling are important in the modulation of neuropathic pain in mice and that these chemokines and their receptors may be interesting targets for future investigations.

## 1. Introduction

For many years, neuropathic pain has remained a challenging issue from a clinical standpoint. Customization of proper treatment for patients is a problem because the mechanism underlying neuropathy is not fully understood [1,2]. Thus, searching for new targets for analgesics is necessary. Currently, many recent studies suggest an important role of nonneuronal cells in the development and maintenance of neuropathic pain. In past studies, alterations in the infiltration, proliferation or activation of microglia, macrophages, neutrophils and astrocytes were confirmed in neuropathy [3,4,5,6,7,8,9,10,11]. The changes were accompanied by alterations in the levels of many chemokines, especially those in the CC subfamily (e.g., CCL1, CCL2, CCL3, CCL9) and their receptors (e.g., CCR2, CCR4, CCR5, CCR8) [6,12,13,14,15,16,17,18]. Additionally, our recent experiments provided the first evidence that both CCR1 and CCR3 are crucial in chronic constriction injury (CCI) of sciatic nerve-evoked neuropathic pain in rats. First, the mRNA levels of some CCR1 and CCR3 ligands are increased after sciatic nerve injury, suggesting their important role not only in chemotaxis but also in nociceptive processes [3,4]. Moreover, we have shown that blockade of CCR1 by J113863 and CCR3 by SB328437 diminished pain-like behaviors, such as mechanical and thermal hypersensitivity, in a rodent CCI model [3,4]. In addition, Kiguchi et al. suggested the involvement of the CCL2/CCR1 and CCL3/CCR1 axes in the development of pain-related symptoms in mice after partial sciatic nerve ligation [19,20]. In our opinion, the role of CCR1 and CCR3 in nociception processes seems to be important but is still poorly understood. Literature data indicate that numerous ligands can act through CCR1 and CCR3, namely, CCL2/3/4/6/9 via CCR1 and CCL11/24/26/28 via CCR3, and importantly, both receptors have three common ligands, namely, CCL5/7/8 [12,21,22,23,24,25]. We found these receptors interesting to study because, to the best of our knowledge, there are only a few studies concerning CCR1 in neuropathy [4,19,20,26,27,28], and there is only one previously published study (by us) [3] on CCR3 involvement in neuropathy.

Therefore, encouraged by previous studies conducted in rats, in the present work, we first studied changes occurring in the whole spectrum of endogenous ligands (CCL2-9/11/24/26/28) and their receptors (CCR1 and CCR3) at the mRNA and/or protein levels in CCI-evoked neuropathic pain by measuring time-dependent changes. Additionally, to define the cellular mechanisms of neuropathy, we studied simultaneous changes in the levels of microglia/macrophages, astrocytes and neutrophils over time. At a later stage, the aim of our research was to determine the pronociceptive properties of chemokines, which are upregulated during neuropathy development. We also sought to assess whether inhibition of selected ligands of these two receptors by bindarit (selective inhibitor of CCL2/7/8 production) can decrease the hypersensitivities evoked by CCI. Moreover, we compared whether blockade of CCR1 with J113863 or CCR3 with SB328437 is more efficient in the treatment of neuropathic pain in a male/female murine model of chronic constriction injury of the sciatic nerve. Additionally, using a dual CCR1/CCR3 antagonist (UCB35625) and by coadministration of J113863 with SB328437, we explored whether simultaneous blockade of the two receptors would exhibit a better analgesic effect than selective blockade. The obtained results extend the knowledge of neuroimmune interactions in neuropathic pain and may contribute to the development of new targets for neuropathic pain treatment in the future.

## 2. Materials and Methods

### 2.1. Animals

Albino Swiss male (16–24 g) and female (20–24 g) mice from Charles River, Germany, were used in the present study. Animals were housed in sawdust-lined cages on a standard 12 h/12 h light/dark cycle (lights on at 06.00 a.m.), with enriched environment, water and food available any time. Experiments were conducted in accordance with the recommendations and standards of the National Institutes of Health Guide for the Care and the International Association for the Study of Pain. Consent to the use of laboratory animals was issued by the Ethical Committee of the Maj Institute of Pharmacology of the Polish Academy of Sciences (213/2021; 75/2017; 97/2022; 1277/2015; 246/2022; 317/2022). Following the 3R policy, the number of animals was reduced to the necessary minimum. Upon arrival at our institute, all mice were subjected to a 5–6 day quarantine. During this time, the animals were habituated using handling techniques. Total amount of mice used in the experiments was: 373 males and 34 females.

### 2.2. Neuropathic Pain Model

**Scheme 1:**
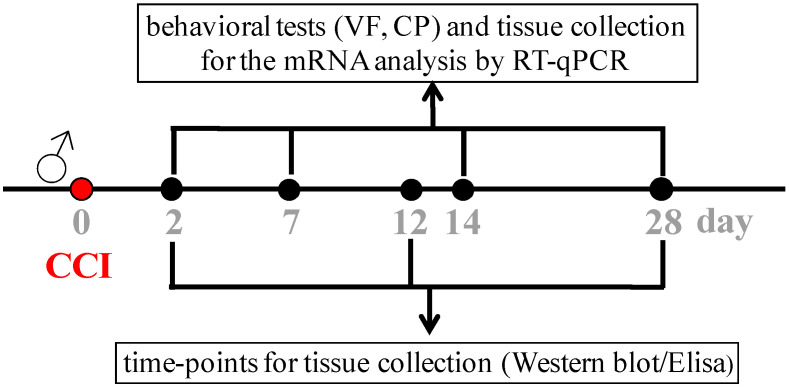
Experiments on Figures 1–3.

Chronic constriction injury of the sciatic nerve in mice was performed under isoflurane anesthesia according to the Bennet and Xie procedure [29]. An incision was made below the right hipbone, and the *biceps femoris* and *gluteus superficialis* were separated. The right sciatic nerve was exposed, and three ligatures (4/0 silk sutures) spaced 1 mm apart were loosely tied around the nerve distal to the sciatic notch until a brief contraction occurred in the operated hind limb. After surgery, mice had at least 2 days for recovery before behavioral measurements (von Frey test (VF), cold plate test (CP), rotarod test (RR)) were performed. For experiments without the use of drugs, behavioral testing and tissue collection were performed according to Scheme 1. Naive mice were chosen as a control for CCI-exposed animals, as in our previously published studies [12,30,31].

### 2.3. Drug Administration

#### 2.3.1. Single Intrathecal Administration

**Scheme 2:**
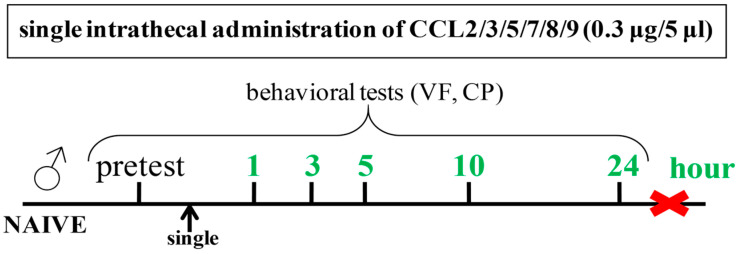
Experiments on Figure 4.

Reconstituted CCL2, CCL3, CCL5, CCL7, CCL8 and CCL9 (Merck, Darmstadt, Germany (CCL8); R&D Systems, Minneapolis, USA (CCL2/3/5/7/8/9)) were dissolved in water for injection and administered once intrathecally (*i.t.*) to naive male mice at a dose of 0.3 μg/5 μL. The control (vehicle) naive group received water for injection (*i.t.*). Intrathecal administrations were performed using the lumbar puncture technique according to Hylden and Wilcox [32], as reported in our previous papers [16,26,30,33,34]. The *i.t*. injections were performed using a Hamilton syringe with a thin needle, and substances were injected into the lumbar part of the spinal cord (between the L5 and L6 vertebrae) in a volume of 5 μL. Behavioral testing was performed according to Scheme 2.

#### 2.3.2. Single Intrathecal Administration

**Scheme 3:**
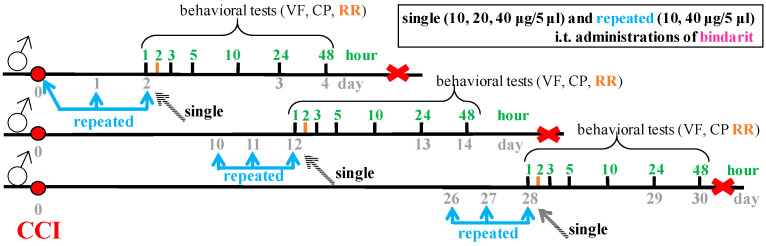
Experiments on Figures 5 and 6.

Bindarit (selective inhibitor of CCL2/CCL7/CCL8 production) (Merck, Darmstadt, Germany) was dissolved in 70% DMSO and administered once *i.t.* to operated animals on the 2nd, 12th and 28th days post CCI at doses of 10, 20, and 40 μg/5 μL. Control animals received 70% DMSO (vehicle, *i.t.*). Intrathecal administrations were performed as described above. Three independent sets of experiments were performed; thus, different groups of animals were used at each time point (early, middle and late phase of neuropathy). Behavioral testing was performed 1, 2, 3, 5, 10, 24 and 48 h after single administration according to Scheme 3.

#### 2.3.3. Repeated Intrathecal Administration

Bindarit (Merck) was dissolved in 70% DMSO and administered repeatedly (3 injections) once per day to operated animals on the following days: (1) 0 (day of CCI surgery), 1st and 2nd days post CCI; (2) 10th, 11th and 12th days post CCI; or (3) 26th, 27th and 28th days post CCI at doses of 10 and 40 μg/5 μL (*i.t*.). Control animals received 70% DMSO (vehicle, *i.t.*) at the same time points. Intrathecal administrations were performed as described above. Three independent sets of experiments were performed; thus, different groups of animals were tested at each time point (early, middle and late phase of neuropathy). Behavioral testing was performed 1, 2, 3, 5, 10, 24 and 48 h after third bindarit administration according to Scheme 3. 

#### 2.3.4. Single Intrathecal Administration

**Scheme 4:**
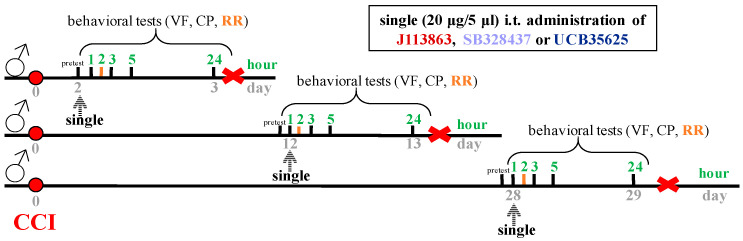
Experiments on Figures 7 and 8.

J113863 (CCR1 antagonist), SB328437 (CCR3 antagonist), and UCB35625 (CCR1/CCR3 antagonist) (all obtained from Tocris, Bristol, UK) were dissolved in 70% DMSO and administered once *i.t.* to operated male animals on the 2nd, 12th and 28th days post CCI at a dose of 20 μg/5 μL. Control animals received 70% DMSO (vehicle, *i.t.*) at the same time points. Intrathecal administrations were performed as described above. Three independent sets of experiments were performed; thus, different groups of animals were tested at each time point (early, middle and late phase of neuropathy). Behavioral testing was performed 1, 2, 3, 5 and 24 h after single drug administration according to Scheme 4. 

#### 2.3.5. Single Intrathecal Administration

**Scheme 5:**
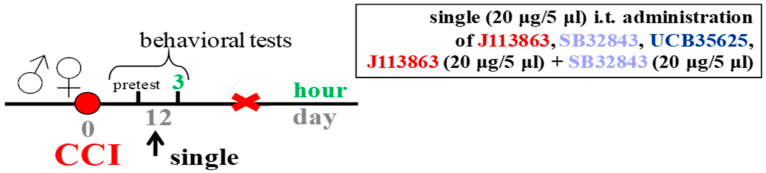
Experiments on Table 3.

J113863, SB328437, UCB35625 and J113863+SB328437 (Tocris) were dissolved in 70% DMSO and administered once *i.t.* to operated male and female animals on the 12th day and post CCI at a dose of 20 μg/5 μL each. Control animals received 70% DMSO (vehicle, *i.t.*) at the same time points. Intrathecal administrations were performed as described above. Behavioral testing was performed 3 h after single drug administration according to Scheme 5.

### 2.4. Behavioral Tests

#### 2.4.1. Mechanical Hypersensitivity Measurement (von Frey Test)

Hypersensitivity to mechanical stimuli in mice was assessed as first, using calibrated nylon monofilaments (Stoelting, Wood Dale, IL, USA). The animals were placed in plastic cages with a wire mesh floor. The hypersensitivity was measured by applying von Frey monofilaments at increasing strength (0.6–6 g (6 g is a cut-off latency)) sequentially to the plantar surface of the hind paw of each mice until the hind paw was withdrawn (e.g., raised or trembled), as described in our previous papers [13,35]. In naive mice, both hind paws were tested. 

#### 2.4.2. Thermal Hypersensitivity Measurement (Cold Plate Test)

Hypersensitivity to thermal stimuli was measured using a cold/hot plate apparatus (Ugo Basile, Gemonio, Italy) as described previously [13,35]. The temperature was maintained at 2 °C, and the cut-off latency was 30 s. Animals were placed on the plate, and the time to lift the right hind paw was recorded. In naive mice, both hind paws were observed. The thermal hypersensitivity assessment was always performed as second, 2–3 min after von Frey test.

#### 2.4.3. Motor Activity Measurement (Rotarod Test)

Animals were placed in separate compartments on a rotating horizontal rod that was accelerated from 2 to 40 rpm, as described previously [30,35]. The time (s) was recorded, when mice fell from the apparatus. The cut-off latency was 300 s. The animals were habituated to the apparatus and trained to walk during handling procedures.

### 2.5. Analysis of Gene Expression

#### Reverse Transcription Quantitative Real-Time Polymerase Chain Reaction

Immediately after decapitation, the dorsal lumbar segments of the spinal cord (L4–L6) were collected from naive and CCI-exposed mice on the 2nd, 7th, 14th and 28th days post CCI, around 30 min after behavioral assessments. Based on Chomczynski and Sacchi studies [36], total RNA was extracted using TRIzol reagent (Invitrogen, Carlsbad, CA, USA). The quality and concentration of RNA were checked using a DeNovix DS-11 spectrophotometer (DeNovix Inc., Wilmington, DE, USA). RNase inhibitor (Promega, Mannheim, Germany), an Omniscript RT Kit (Qiagen Inc., Hilden, Germany) and oligo (dT16) primer (Qiagen Inc., Hilden, Germany) were used to perform reverse transcription of 1 μg of total RNA at 37 °C. Next, the obtained cDNA templates were diluted 1:10 using RNase-/DNase-free H_2_O. RT–qPCR was performed with about 50 ng of cDNA templates from each sample using Assay-On-Demand TaqMan probes (Applied Biosystems, Foster City, CA, USA) and an iCycler device (Bio-Rad, Hercules, Warsaw, Poland). The following TaqMan primers were used: *CCL2* (Mm00441243_g1), *CCL3* (Mm00441258_m1), *CCL4* (Mm00443112_m1), *CCL5* (Mm01302428_m1), *CCL6* (Mm01302419_m1), *CCL7* (Mm01308393_g1), *CCL8* (Mm01297183_m1), *CCL9* (Mm00441262_g1), *CCL11* (Mm00441238_m1), *CCL24* (Mm00444701_m1), *CCL26* (Mm04204096_m1), *CCL28* (Mm00445039_m1), *IBA-1* (Mm01132448_g1), *GFAP* (Mm00546086_m1), *MPO* (Mm01298424_m1), *CD8* (Mm01182108_m1), *CD4* (Mm00442758_g1), and *HPRT* (Mm00446968_m1). *HPRT* was used as an endogenous control and an adequate housekeeping gene. The cycle threshold values were automatically calculated using CFX Manager v.2.1 software (Bio-Rad, Warsaw, Poland) with the default parameters. The RNA content was calculated using the formula 2^−(threshold cycle)^.

### 2.6. Analysis of Protein Levels

#### 2.6.1. Western Blotting

Immediately after decapitation, the tissues from ipsilateral spinal cord segments (L4–L6) were collected from naive and CCI-exposed mice on the 2nd, 12th and 28th days post CCI, around 30 min after behavioral assessments. The samples were then placed in RIPA buffer supplemented with a protease inhibitor cocktail (Sigma-Aldrich, St. Louis, MO, USA), homogenized and cleared via centrifugation (14,000 rpm, 30 min 4 °C). The total protein concentration was checked using the bicinchoninic acid method. The obtained samples (10 µg of protein) were heated in a mix of loading buffer (4x Laemmli Buffer, Bio-Rad, Warsaw, Poland) and 2-mercaptoethanol (Bio-Rad) for 8 min at 98 °C. Using 4–15% Criterion™ TGX™ precast polyacrylamide gels (Bio-Rad), electrophoresis was performed. Next, the proteins were transferred (semidry transfer 25 V, 30 min) to Immune-Blot PVDF membranes (Bio-Rad) and next blocked for 1 h at RT using 5% bovine serum albumin (Sigma-Aldrich) in Tris-buffered saline containing 0.1% Tween-20 (TBST). Following transfer, the membranes were washed with TBST and incubated overnight (4 °C) with the following primary antibodies: rabbit: anti-CCR1 (1:1000 Novus, NBP1-78173; Abingdon, UK), anti-CCR3 (1:1000, NBP1-77065; Novus), anti-IBA-1 (1:500, NBP2-19019; Novus), anti-GFAP (1:10 000, NB300-141; Novus), anti-MPO (1:1000, ab208670; Abcam, Cambridge, UK); mouse: anti-GAPDH (1:5000, MAB374; Merck, Darmstadt, Germany). Afterward, the membranes were washed with TBST and incubated for 1 h at room temperature with HRP-conjugated anti-rabbit or anti-mouse secondary antibodies (1:5000, Vector Laboratories, Burlingame, CA, USA) diluted in a SignalBoost™ Immunoreaction Enhancer Kit (Merck) buffer. Detection of protein was obtain using Clarity™ Western ECL Substrate (Bio-Rad) and visualized using a Fujifilm LAS-4000 FluorImager system. To estimate the intensities of immunoreactive bands, Fujifilm MULTI GAUGE software V3.0 (Tokio, Japan) was used.

#### 2.6.2. Enzyme-Linked Immunosorbent Assay

Samples for protein analysis were prepared for measurements as described above. ELISAs for CCL2 (ABclonal, RK00381 Woburn, MA, USA), CCL3 (LSBio, LS–F4952; Seattle, WA, USA), CCL4 (LSBio, LS-F4954), CCL5 (ABclonal, RK00167), CCL6 (Abbexa, abx353305; Cambridge, UK), CCL7 (ABclonal, RK06183), CCL8 (ABclonal, RK00425) and CCL9 (LSBio, LS-F55161) were performed according to the manufacturer’s instructions. The detection limits were as follows: CCL2, 3.12–1000 pg/mL; CCL3, 15.6–1000 pg/mL; CCL4, 12.5–800 pg/mL; CCL5, 39–2500 pg/mL; CCL6, 78–5000 pg/mL; CCL7, 15.6–1000 pg/mL; CCL8, 78–10,000 pg/mL; and CCL9, 7.8–500 pg/mL. Positive controls for testing were provided by the manufacturers.

### 2.7. Statistical Analysis

**Figure 1:**
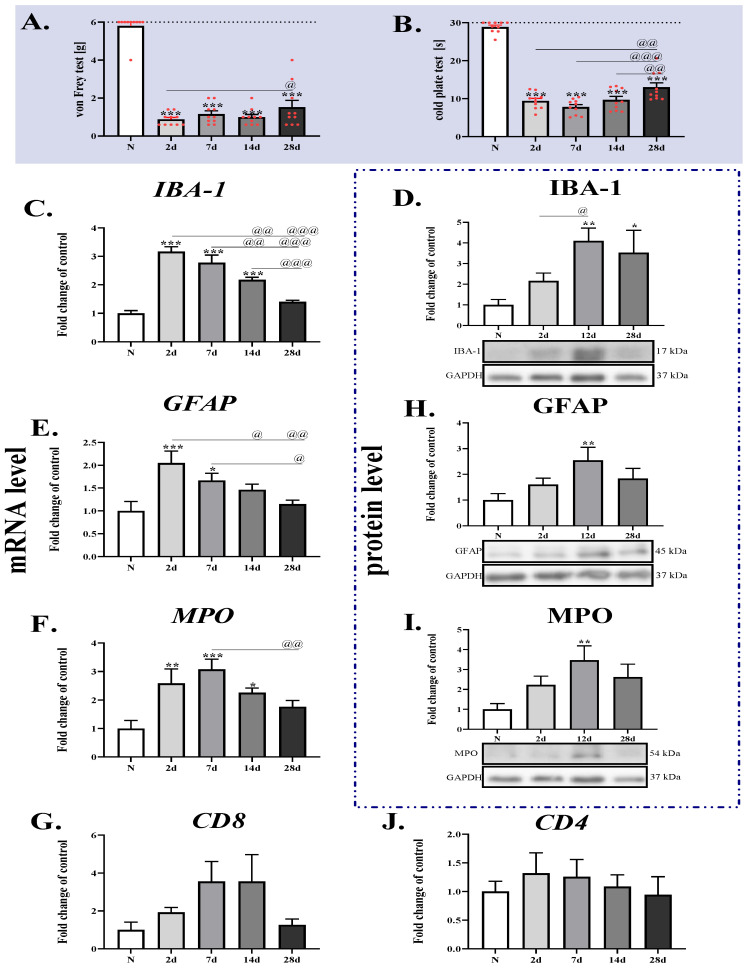
Time-dependent changes in mechanical and thermal hypersensitivity in male mice; (**A**) von Frey test; (**B**) cold plate test and associated changes in IBA-1, GFAP, MPO, CD8 and CD4 mRNA ((**C**,**E**,**F**,**G**,**J**), respectively) and protein IBA-1, GFAP, MPO ((**D**,**H**,**I**), respectively) levels in the spinal cord on the 2nd, 7th, 12th, 14th and 28th days after chronic constriction injury of the sciatic nerve. The behavioral data are presented as the means ± SEMs. The red dots indicate individual measurements. The horizontal dotted line shows the cut off value. The RT–qPCR or Western blot data are presented as the fold change of control ± SEMs. Intergroup differences were analyzed using one-way ANOVA with Bonferroni’s post hoc test for multiple comparisons. * *p* < 0.05, ** *p* < 0.01, and *** *p* < 0.001 indicate a significant difference compared with the control group (naive). @ *p* < 0.05, @@ *p* < 0.01, and @@@ *p* < 0.001 indicate differences between the 2nd vs. 7th, 2nd vs. 12th, 2nd vs. 14th, 2nd vs. 28th, 7th vs. 14th, 7th vs. 28th, 12th vs. 28th, and 14th vs. 28th days. The quantity of animals used in the experiment was as follows: Behavioral tests: N (*n* = 10), 2 d (*n* = 10), 7 d (*n* = 10), 14 d (*n* = 10), 28 d (*n* = 10); RT-qPCR: N (*n* = 4–6), 2 d (*n* = 8), 7 d (*n* = 8–9), 14 d (*n* = 10), 28 d (*n* =9); Western blot: N (*n* = 5–6), 2 d (*n* = 5–6), 12 d (*n* = 6), 28 d (*n* = 5). Abbreviations: N—naive.

Data from behavioral experiments (Figures 1A,B, 4A,B, 5A–F, 6A–F, 7A,C,E and 8A,C,E and Tables 1 and 2) are shown as mean grams or seconds ± standard error of the mean (SEM). Intergroup differences were analyzed using one-way analysis of variance (ANOVA) followed by Bonferroni’s post hoc test for multiple comparisons. In Figure 1A,B, the individual data points are shown. Moreover, area under the curve (AUC) was calculated to compare the effects of the tested compounds (Figures 4C,D, 7B,D,F and 8B,D,F). Additionally, the results were evaluated using two-way ANOVA, (Supplementary S1) to determine the time × drug or sex × drug interactions (Figures 4A,B, 5A–F, 6A–F, 7A,C,E and 8A,C,E and Table 3) and *t* test (Table 3). Moreover, the % maximum possible effect (%MPE) was calculated as the percentage difference between the measured response and the baseline response, divided by the difference between the maximum response and the baseline response (Table 3). For biochemical analyses, the RT–qPCR, ELISA and Western blotting results are presented as the fold change relative to the control (naive) ± SEM. The number of animals was chosen based on our previous research [30,31,37]. Similar to the behavioral studies, the data were analyzed using one-way analysis of variance followed by Bonferroni’s post hoc test. Obtained data were analyzed using GraphPad Prism 9 software (GraphPad, San Diego, CA, USA).

## 3. Results

### 3.1. Time Course of Changes in the Pain-Related Behaviors and mRNA or Protein Levels of IBA-1, GFAP, MPO, CD4, and CD8 in the Spinal Cord Measured on the 2nd, 7th, 12th, 14th and/or 28th Day after Chronic Constriction Injury of the Sciatic Nerve in Mice

Sciatic nerve injury resulted in the development of mechanical (F = 95.99, *p* < 0.0001; Figure 1A) and thermal (F = 115.60, *p* < 0.0001; Figure 1B) hypersensitivity between the 2nd and 28th days post CCI. The behavioral changes were accompanied by elevated mRNA levels of IBA-1 and MPO between the 2nd and 14th day (F = 29.09, *p* < 0.0001, Figure 1C; F = 5.42, *p* = 0.0015, Figure 1F) and GFAP until the 7th day (F = 5.68, *p* = 0.0011, Figure 1E). No significant changes were observed for CD8 (Figure 1G) or CD4 (Figure 1J) at the selected time points.

At the protein level, upregulation of the IBA-1 (F = 4.64, *p* = 0.0142, Figure 1D), GFAP (F = 3.28, *p* = 0.0437, Figure 1H) and MPO (F = 3.27, *p* = 0.0469, Figure 1I) levels was observed on the 12th day after CCI. Moreover, on the 28th day post CCI, the IBA-1 protein level was still upregulated.

### 3.2. Time Course of Changes in the mRNA Levels of CCL2, CCL3, CCL4, CCL5, CCL6, CCL7, CCL8, CCL9, CCL11, CCL24, CCL26, and CCL28 in the Spinal Cord Measured on the 2nd, 7th, 14th and 28th Days after Chronic Constriction Injury of the Sciatic Nerve in Mice

**Figure 2:**
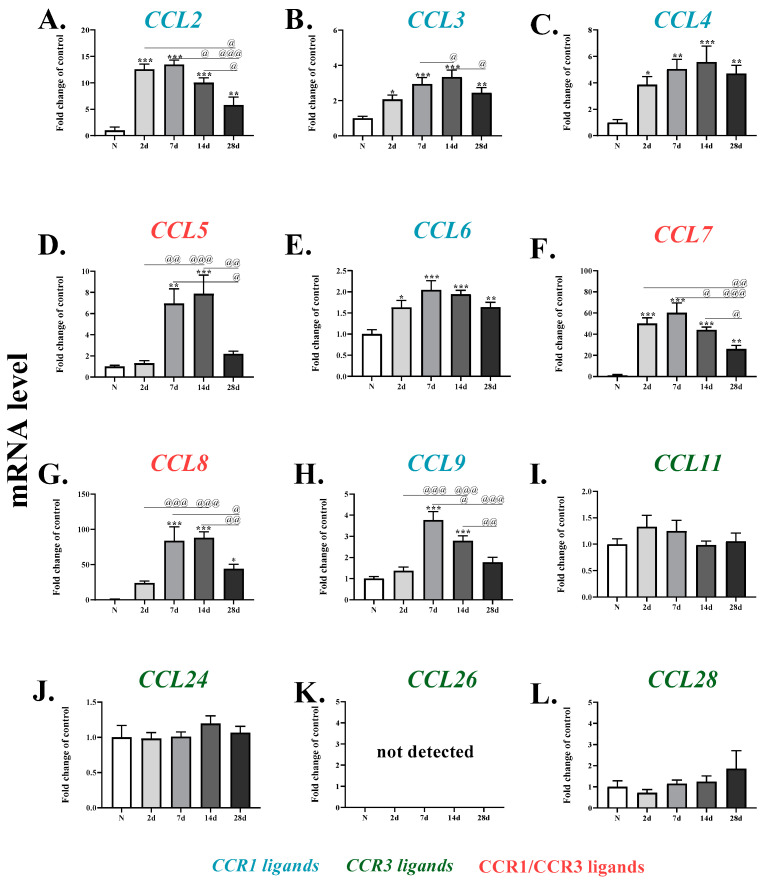
Time-dependent changes in CCL2, CCL3, CCL4, CCL5, CCL6, CCL7, CCL8, CCL9, CCL11, CCL24, CCL26 and CCL28 mRNA levels (**A**–**L**) in male mice on the 2nd, 7th, 14th and 28th days after chronic constriction injury of the sciatic nerve in the spinal cord. The RT–qPCR data are presented as the fold change of control ± SEMs. Intergroup differences were analyzed using one-way ANOVA with Bonferroni’s post hoc test for multiple comparisons. * *p* < 0.05, ** *p* < 0.01, and *** *p* < 0.001 indicate a significant difference compared with the control group (naive). @ *p* < 0.05, @@ *p* < 0.01, and @@@ *p* < 0.001 indicate differences between the 2nd vs. 7th, 2nd vs. 14th, 2nd vs. 28th, 7th vs. 14th, 7th vs. 28th, and 14th vs. 28th days. The quantity of animals used in the experiment was as follows: RT-qPCR: N (*n* = 5–7), 2d (*n* = 8), 7d (*n* = 8–10), 14d (*n* = 10), 28d (*n* = 8–9). Abbreviations: N—naive.

The most upregulated chemokine compared to vehicle was CCL8 (F = 10.74, *p* < 0.0001, Figure 2G), whose elevated level lasted from the 7th to the 28th day post CCI. Likewise, high levels after surgery were observed for CCL7 (F = 15.44, *p* < 0.0001, Figure 2F), and these changes lasted until the last day of the experiment. Another strongly elevated chemokine was CCL2 (F = 19.14, *p* < 0.0001, Figure 2A), which was significantly elevated until the 28th day post CCI. Lower but still significant upregulation was observed for CCL3 (F = 7.32, *p* = 0.0002, Figure 2B), CCL4 (F = 4.28; *p* = 0.0057, Figure 2C) and CCL6 (F = 6.23, *p* = 0.0006, Figure 2E), and the changes were observed at all time points in the experiment. In the case of CCL5 (F = 7.92, *p* = 0.0001, Figure 2D) and CCL9 (F = 17.10, *p* < 0.0001, Figure 2H), changes in their levels started to appear on the 7th day and lasted until the 14th day post CCI. No significant changes occurred for CCL11 (Figure 2I), CCL24 (Figure 2J) or CCL28 (Figure 2L). Moreover, CCL26 mRNA (Figure 2K) was not detected in the mouse spinal cord.

### 3.3. Time Course of Changes in the Protein Levels of CCR1, CCR3, CCL2, CCL3, CCL4, CCL5, CCL6, CCL7, CCL8, and CCL9 in the Spinal Cord Measured on the 2nd, 12th and 28th Days after Chronic Constriction Injury of the Sciatic Nerve in Mice

**Figure 3:**
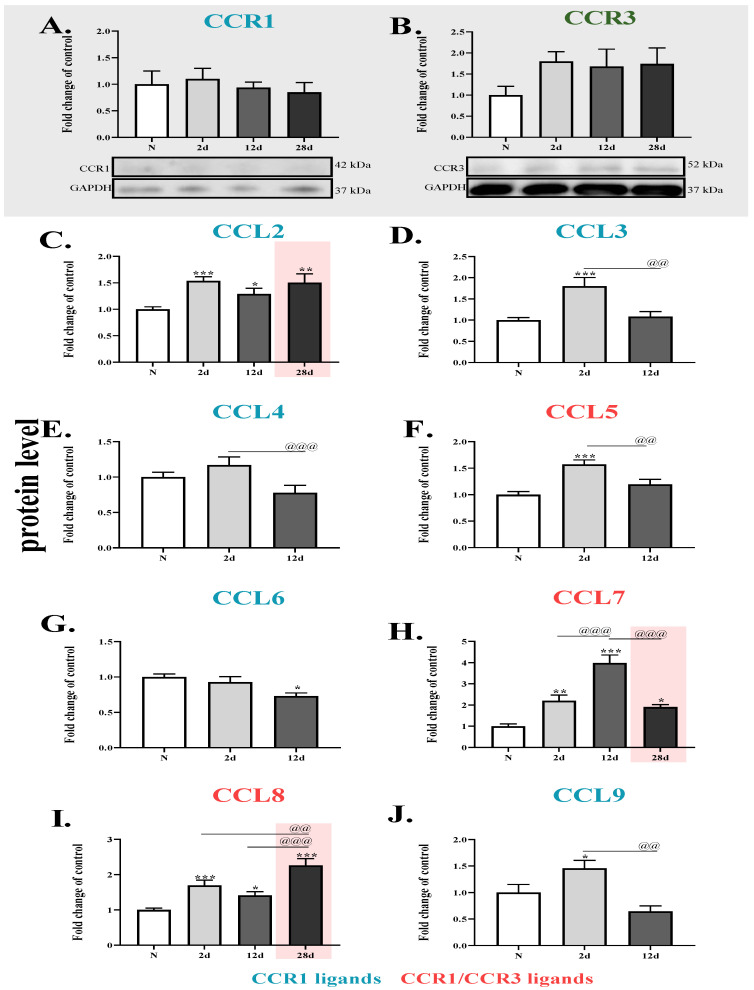
Time-dependent changes in CCR1, CCR3, CCL2, CCL3, CCL4, CCL5, CCL6, CCL7, CCL8, and CCL9 protein levels (**A**–**J**) in male mice on the 2nd, 12th and 28th days after chronic constriction injury of the sciatic nerve in the spinal cord. The Western blot/ELISA data are presented as the fold change of control ± SEMs. Intergroup differences were analyzed using one-way ANOVA with Bonferroni’s post hoc test for multiple comparisons. * *p* < 0.05, ** *p* < 0.01, and *** *p* < 0.001 indicate a significant difference compared with the control group (naive). @@ *p* < 0.01 and @@@ *p* < 0.001 indicate differences between the 2nd vs. 12th, 2nd vs. 28th, and 12th vs. 28th days. The quantity of animals used in the experiment was as follows: Western blot: N (*n* = 5–6), 2d (*n* = 6), 12d (*n* = 6), 28d (*n* = 5–6); ELISA: N (*n* = 6), 2d (*n* = 5–6), 12d (*n* = 5–6), 28d (*n* = 4–5). Abbreviations: N—naive.

Sciatic nerve surgery did not lead to upregulation of CCR1 and CCR3 between the 2nd and 28th day post CCI (Figure 3A,B). Among all the tested chemokines, there was significant upregulation of three of them, namely, CCL2 (F = 6.75, *p* = 0.0034, Figure 3C), CCL7 (F = 25.32, *p* < 0.0001, Figure 3H) and CCL8 (F = 16.04, *p* < 0.0001, Figure 3I), observed at all tested time points between the 2nd and 28th day post CCI. Enhanced levels of CCL3 (F = 10.04, *p* = 0.0017, Figure 3D), CCL5 (F = 13.19, *p* = 0.0005, Figure 3F) and CCL9 (F = 8.08, *p* = 0.0052, Figure 3J) were detected 2 days after sciatic nerve surgery. On the 12th day, the CCL6 (F = 5.95, *p* = 0.0125, Figure 3G) level was slightly downregulated compared to that in naive mice. No differences were observed between naive and CCI animals in the case of CCL4; however, changes occurred between the 2nd and 12th days after surgery (F = 3.97, *p* = 0.0413, Figure 3E).

### 3.4. Effects of a Single i.t. CCL2, CCL3, CCL5, CCL7, CCL8 and CCL9 Administration on Mechanical and Thermal Hypersensitivity Measured in Naive Mice

**Figure 4:**
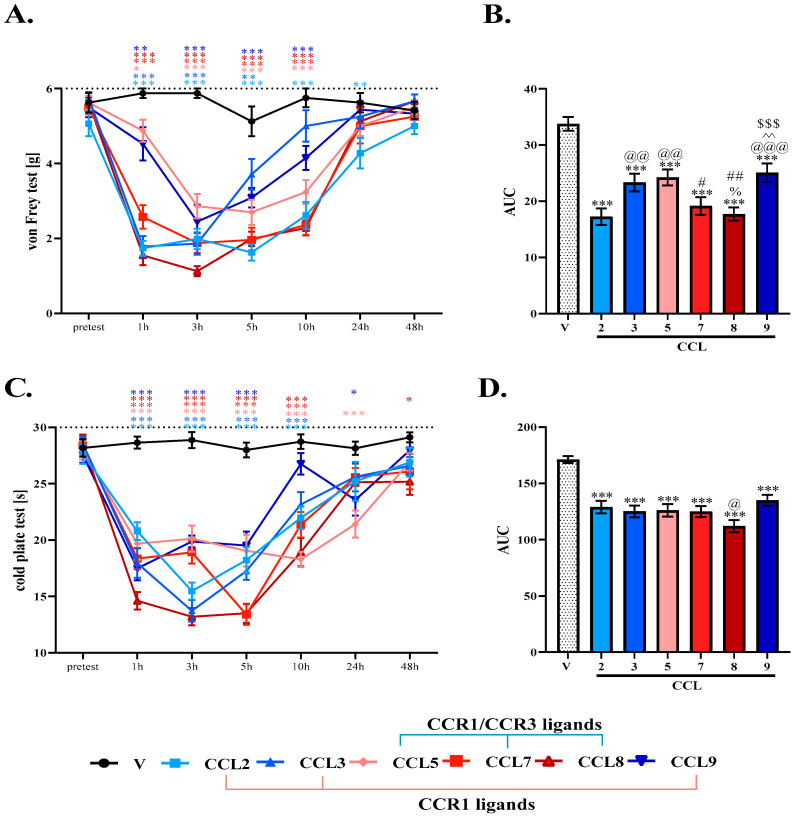
Effect of a single (0.3 μg/5 μL) *i.t.* administration of CCL2, CCL3, CCL5, CCL7, CCL8 and CCL9 on mechanical (A) and thermal hypersensitivity (C) in male mice as measured 1, 3, 5, 10, 24 and 48 h after injection. Moreover, the obtained data were analyzed as areas under the curve (**B**,**D**). The data are presented as the means ± SEMs (**A**,**C**) or total area under curve ± SEMs. Intergroup differences were analyzed using one-way ANOVA with Bonferroni’s post hoc test for multiple comparisons. * *p* < 0.05, ** *p* < 0.01, and *** *p* < 0.001 indicate a significant difference compared with the control group. @ *p* < 0.05, @@ *p* < 0.01, and @@@ *p* < 0.001 indicate differences between the CCL2 group; % *p* < 0.05 indicates differences between the CCL3 group; # *p* < 0.05, and ## *p* < 0.01 indicate differences between the CCL5 group; ^^ *p* < 0.01 indicates differences between the CCL7 group; $$$ *p* < 0.001 indicates differences between the CCL8 group. The quantity of animals used in the experiment was as follows: V (*n* = 8), CCL2 (*n* = 8), CCL3 (*n* = 8), CCL5 (*n* = 7–8), CCL7 (*n* = 8), CCL8 (*n* = 8), CCL9 (*n* = 7–8). Abbreviations: V—control, 2/3/5/7/8/9—CCL2/3/5/7/8/9.

A single intrathecal injection of CCL2, CCL3, CCL5, CCL7, CCL8 and CCL9 induced mechanical (Figure 4A) and thermal (Figure 4C) hypersensitivity. 

Painful reactions were observed after 1 h and lasted until 5 h for all examined chemokines in both tests. Moreover, in the von Frey test, the CCL2 effect was observed until 24 h postinjection. In the case of cold plates, CCL5 and CCL9 maintained their effect until 24 h after injection. A slight pronociceptive effect was observed in the same test for CCL8 48 h post administration. The strongest pain-related reaction in the von Frey and cold plate tests was observed for CCL8 3 h after injection (F = 29.04, *p* < 0.0001; F = 38.24, *p* < 0.0001; Figure 4A,C, respectively). However, analysis of the area under the curve revealed that CCL2 was the most pronociceptive chemokine, taking into consideration all time points, in the case of mechanical hypersensitivity (F = 15.37, *p* < 0.0001; Figure 4B), while in the case of thermal hypersensitivity (F = 13.72, *p* < 0.0001; Figure 4D), the most pain-evoking chemokine was CCL8. Moreover, two-way ANOVA confirmed a significant interaction between the treatment and the analyzed time points in the von Frey test (F = 8.74; *p* < 0.0001) and cold plate test (F = 6.57; *p* < 0.0001). Post hoc comparisons for this analysis are available in Supplementary S1.

### 3.5. Effects of Single and Repeated i.t. Bindarit Administration on Mechanical Hypersensitivity Measured 2, 12 and 28 Days after Chronic Constriction Injury in Mice

**Figure 5:**
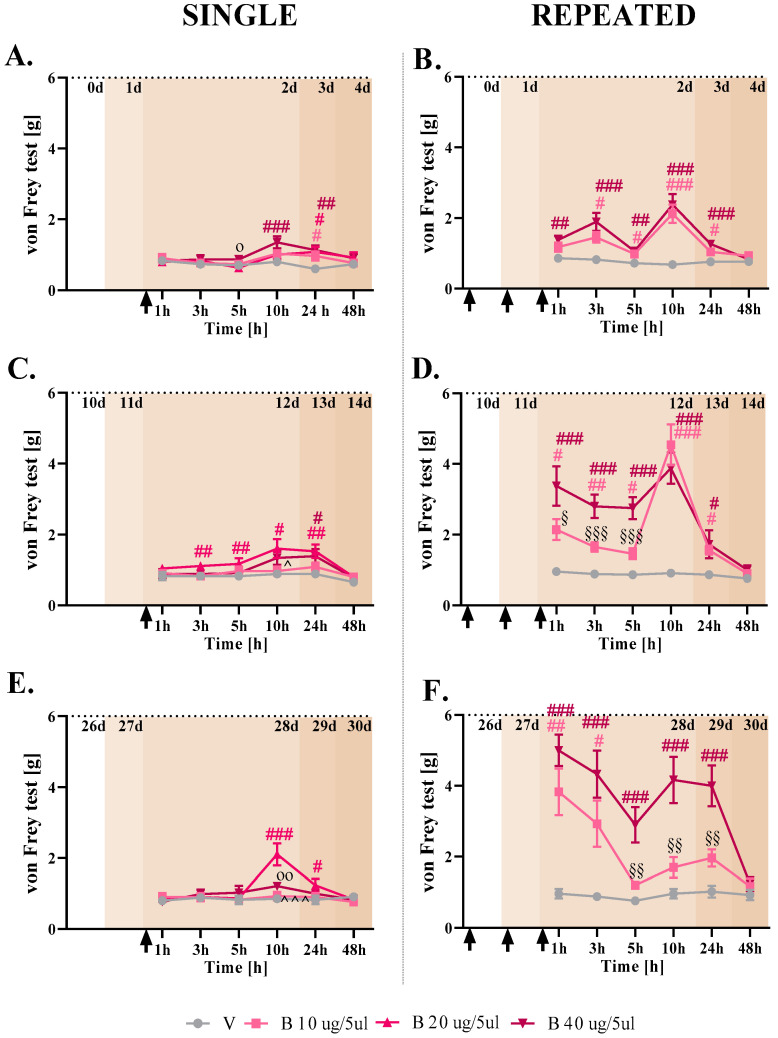
Effect of a single (10, 20 and 40 μg/5 μL; (**A**,**C**,**E**)) and repeated (10 and 40 μg/5 μL; (**B**,**D**,**F**)) *i.t.* administration of bindarit on mechanical hypersensitivity in male mice as measured on the 2nd, 12th, and 28th days after chronic constriction injury of the sciatic nerve. Measurements were performed 1, 3, 5, 10, 24 and 48 h after a single drug administration or after three doses of the given substance. The horizontal dotted line shows the cutoff value. The data are presented as the means ± SEMs. Intergroup differences were analyzed using one-way ANOVA with Bonferroni’s post hoc test for multiple comparisons. # *p* < 0.05, ## *p* < 0.01, and ### *p* < 0.001 indicate a significant difference compared with the vehicle-treated group. ^ *p* < 0.05 and ^^^ *p* < 0.001 indicate a significant difference between B 10 and 20 μg/5 μL; § *p* < 0.05, §§ *p* < 0.01 and §§§ *p* < 0.001 indicate a significant difference between B 10 and 40 μg/5 μL; ᴏ *p* < 0.05 and ᴏᴏ *p* < 0.01 indicate a significant difference between B 20 and 40 μg/5 μL. The quantity of animals used in the experiment was as follows: Single injection: (2nd day: V (*n* = 6), B10 (*n* = 6), B20 (*n* = 6), B40 (*n* = 6); 12th day: V (*n* = 7), B10 (*n* = 7), B20 (*n* = 7), B40 (*n* = 7); 28th day: V (*n* = 7), B10 (*n* = 6), B20 (*n* = 6), B40 (*n* = 7)); Repeated injection: (2nd day: V (*n* = 10), B10 (*n* = 10), B40 (*n* = 10); 12th day: V (*n* = 8–9), B10 (*n* = 8), B40 (*n* = 7); 28th day: V (*n* = 5), B10 (*n* = 6), B40 (*n* = 6)). Abbreviations: B—Bindarit, ↑—injection.

As shown in Figure 1, sciatic nerve surgery led to the development of mechanical hypersensitivity in mice, which was observed between the 2nd and 28th day. Behavioral tests on the 2nd day showed that a single bindarit administration at the highest dose of 40 µg/5 µL slightly reduced mechanical hypersensitivity between 10 and 24 h after injection; however, the strongest effect was obtained after 10 h (F = 3.73, *p* = 0.0348, Figure 5A). All tested doses revealed slight analgesic effects 24 h after substance administration. In comparison, repeated administration of bindarit on the 2nd day post CCI was far more effective than a single administration. Reduction of hypersensitivity was observed just after the third injection of bindarit and lasted until 24 h post injection. The highest bindarit effectiveness was observed 10 h after the third injection of 40 µg/5 µL (F = 12.18, *p* < 0.0001, Figure 5B).

The situation was similar at the later time points. As measured 12 days after sciatic nerve surgery, repeated administration of bindarit was much more effective than a single injection. Moreover, after repeated injections, similar to the 2nd day post CCI, the highest bindarit effectiveness was obtained 10 h after the third injection of the 10 µg/5 µL dose (F = 19.15, *p* < 0.0001, Figure 5D). In the case of a single administration, the most effective dose was 20 µg/5 µL, measured 10 h after administration (F = 3.36, *p* = 0.0353, Figure 5C).

On the last (28th) day of the experiment, after a single injection, the only effective dose was 20 µg/5 µL, with the peak of action 10 h post injection (F = 10.0, *p* = 0.0002, Figure 5E), whereas after repeated administration, the most effective bindarit dose was 40 µg/5 µL, measured 1 h after the third injection (F = 16.88, *p* = 0.0002, Figure 5F), and its duration of action reached 24 h after injection. Moreover, two-way ANOVA confirmed a significant interaction after single injection between the treatment and the analyzed time points in the von Frey test 28 days post CCI (F = 3.21; *p* = 0.0002), while no interaction was observed on the 2nd and 12thday. In the case of repeated administration, interactions were confirmed in all tested time points (2nd day, F = 5.04; *p* < 0.0001, 12th day F = 7.12; *p* < 0.0001, 28th day F = 3.18; *p* = 0.0017). Post hoc comparisons for this analysis are available in Supplementary S1.

### 3.6. Effects of Single and Repeated i.t. Bindarit Administration on Thermal Hypersensitivity Measured 2, 12 and 28 Days after Chronic Constriction Injury in Mice

**Figure 6:**
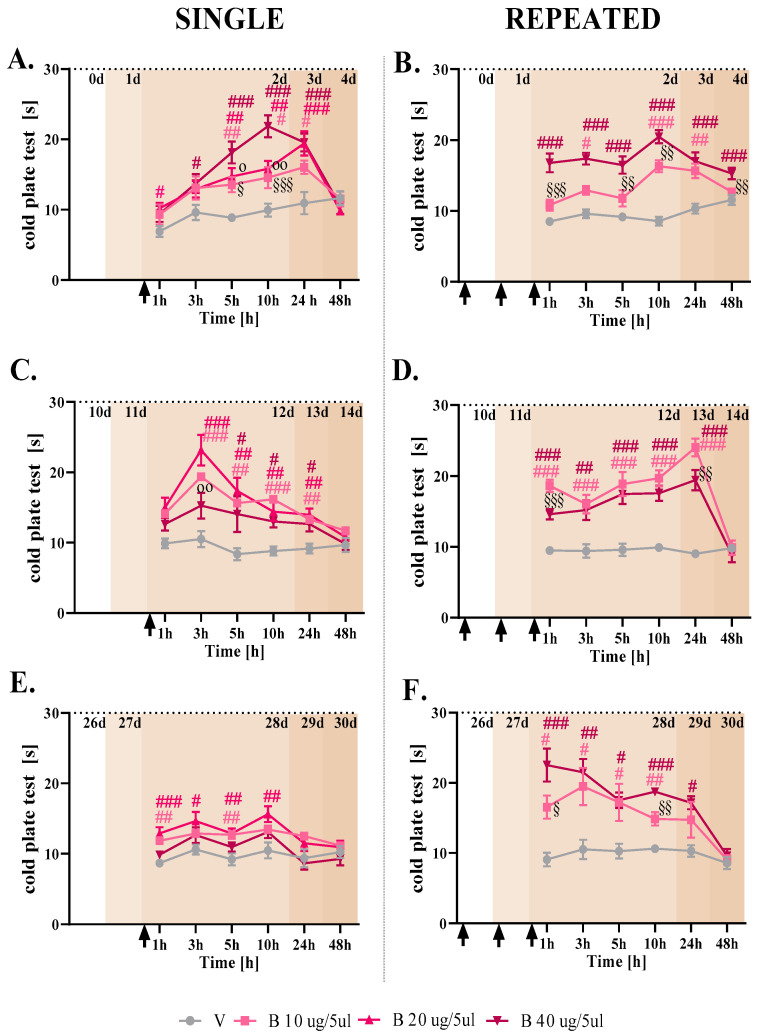
Effect of a single (10, 20 and 40 μg/5 μL; (**A**,**C**,**E**)) and repeated (10 and 40 μg/5 μL; (**B**,**D**,**F**)) *i.t.* administration of bindarit on thermal hypersensitivity in male mice as measured on the 2nd, 12th and 28th days after chronic constriction injury of the sciatic nerve. Measurements were performed 1, 3, 5, 10, 24 and 48 h after a single drug administration or after three doses of the given substance. The horizontal dotted line shows the cutoff value. The data are presented as the means ± SEMs. Intergroup differences were analyzed using one-way ANOVA with Bonferroni’s post hoc test for multiple comparisons. # *p* < 0.05, ## *p* < 0.01, and ### *p* < 0.001 indicate a significant difference compared with the vehicle-treated group. § *p* < 0.05, §§ *p* < 0.01 and §§§ *p* < 0.001 indicate a significant difference between B 10 and 40 μg/5 μL; ᴏ *p* < 0.05 and ᴏᴏ *p* < 0.01 indicate a significant difference between B 20 and 40 μg/5 μL. The quantity of animals used in the experiment was as follows: Single injection: (2nd day: V (*n* = 6), B10 (*n* = 6), B20 (*n* = 5, 6), B40 (*n* = 6); 12th day: V (*n* = 7), B10 (*n* = 7), B20 (*n* = 7), B40 (*n* = 6-7); 28th day: V (*n* = 7), B10 (*n* = 6), B20 (*n* = 6), B40 (*n* = 7)); Repeated injection: (2nd day: V (*n* = 10), B10 (*n* = 10), B40 (*n* = 10); 12th day: V (*n* = 8–9), B10 (*n* = 10), B40 (*n* = 8); 28th day: V (*n* = 5), B10 (*n* = 6), B40 (*n* = 6)). Abbreviations: B—Bindarit, V—vehicle, ↑—injection.

As shown in Figure 1, sciatic nerve surgery led to the development of thermal hypersensitivity in mice, which was observed between the 2nd and 28th day. A single administration of bindarit resulted in the development of analgesia, which was detected at three different bindarit doses. On the 2nd day, the strongest antinociceptive effect was obtained 10 h after injection at a dose of 40 µg/5 µL (F = 14.23, *p* < 0.0001, Figure 6A). After repeated administration, we observed a similar level of analgesia; however, after three injections of 40 µg/5 µL bindarit, the reduction in hypersensitivity was maintained even 48 h later (F = 8.47, *p* = 0.0015, Figure 6B).

On the 12th day after surgery, all tested doses exhibited analgesic properties after single and repeated injections. The strongest effect of a single administration was shown for the 20 µg/5 µL dose and was observed 3 h after injection (F = 10.13, *p* = 0.0001, Figure 6C). In the case of repeated administration, the most effective dose of 10 µg/5 µL had the strongest effect 24 h after injection (F = 47.27, *p* < 0.0001, Figure 6D).

On the last (28th) day of the experiment, a single injection of bindarit was only effective at two doses, 10 and 20 µg/5 µL, and the strongest analgesia was observed 10 h after injection of 20 µg/5 µL bindarit (F = 4.36, *p* = 0.0155, Figure 6E). In the case of repeated treatment, the most antinociceptive dose was 40 µg/5 µL, and the peak of action was obtained 1 h after the third injection (F = 12.97, *p* = 0.0006, Figure 6F). Additionally, two-way ANOVA confirmed a significant interaction between the treatment and the analyzed time points in the cold test 2 and 12 days post CCI in case of ingle injection (F = 3.31, *p* = 0.0001; F = 1.76, *p* = 0.046), while no interaction was observed on the day 28th. In the case of repeated administration, interactions were confirmed in all tested time points (2nd day, F = 3.36, *p* = 0.0005; 12th day F = 5.71, *p* < 0.0001; 28th day F = 1.96, *p* = 0.048). Post hoc comparisons for this analysis are available in Supplementary S1.

The sciatic nerve surgery led to the development of disorders in motor coordination. The measurements in a rotarod apparatus for naive mice are in the range from 262.17 s ± 9.90 to 281.17 s ± 11.97 (data not shown in the table); thus, the results observed in the V-treated CCI-exposed mice 2 and 28 days post injury indicate severe motor function disorders (Table 1). Importantly, the single and repeated administrations of bindarit, at any of the tested doses, do not disturb the motor dysfunction observed in V-treated CCI-exposed mice, as measured by the rotarod test on the 2nd, 12th and 28th days after chronic constriction injury of the sciatic nerve (Table 1).

### 3.7. Effects of a Single i.t. J113863, SB328437 and UCB35625 Administration on Mechanical and Thermal Hypersensitivity Measured 2, 12 and 28 Days after Chronic Constriction Injury in Mice

**Figure 7:**
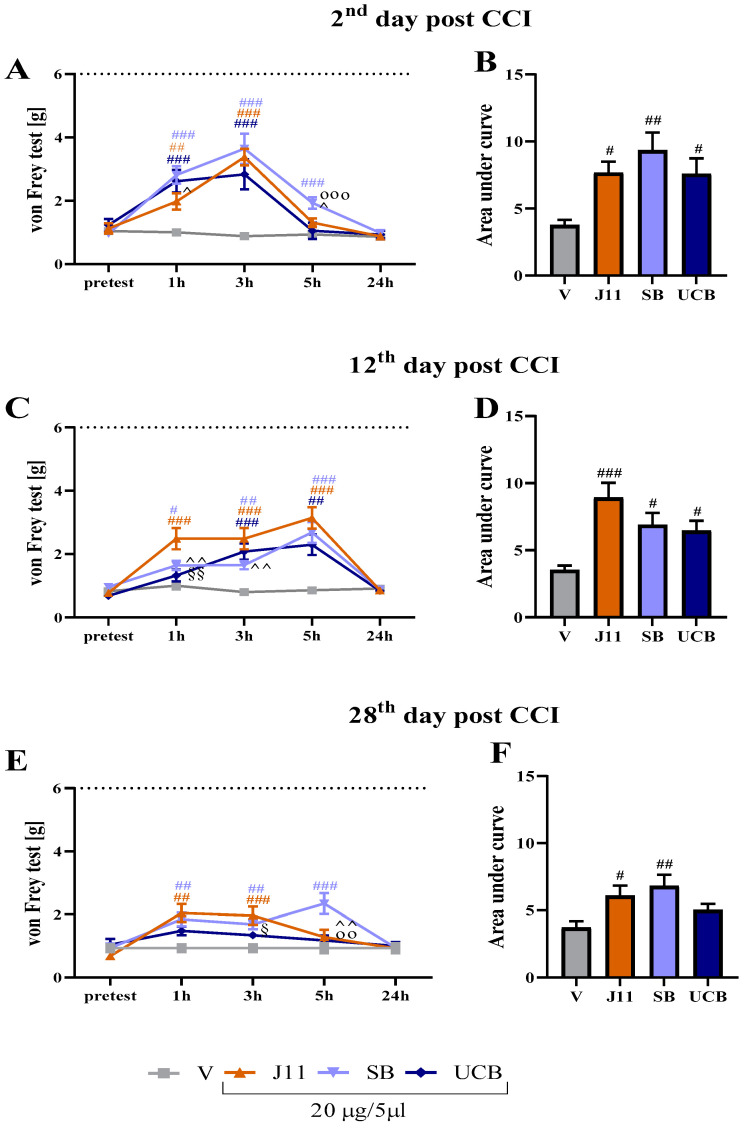
Effect of a single (20 μg/5 μL) *i.t.* administration of J113863, SB328437 or UCB35625 on mechanical hypersensitivity (**A**,**C**,**E**) in male mice as measured on the 2nd, 12th, and 28th days after chronic constriction injury of the sciatic nerve. Measurements were performed 1, 3, 5 and 24 h after substance injection. The horizontal dotted line shows the cut off value. Moreover, the data obtained were analyzed as areas under the curve (**B**,**D**,**F**). The data are presented as the means ± SEMs (**A**,**C**,**E**) or total area under curve ± SEMs (**B**,**D**,**F**). Intergroup differences were analyzed using one-way ANOVA with Bonferroni’s post hoc test for multiple comparisons. # *p* < 0.05, ## *p* < 0.01, and ### *p* < 0.001 indicate a significant difference compared with the vehicle-treated group. ^ *p* < 0.05 and ^^ *p* < 0.001 indicate differences between J11 and SB, § *p* < 0.05, §§ *p* < 0.001 indicate a difference between J11 and UCB; ᴏᴏ *p* < 0.01, ᴏᴏᴏ *p* < 0.001 indicate a significant difference between SB and UCB. The quantity of animals used in the experiment was as follows: 2nd day: V (*n* = 10), J11 (*n* = 7–8), SB (*n* = 10), UCB (*n* = 6); 12th day: V (*n* = 7), J11 (*n* = 7), SB (*n* = 10), UCB (*n* = 5); 28th day: V (*n* = 6), J11 (*n* = 5), SB (*n* = 7), UCB (*n* = 5). Abbreviations: J11—J113863, SB—SB328437, UCB—UCB35625, V—vehicle.

**Figure 8:**
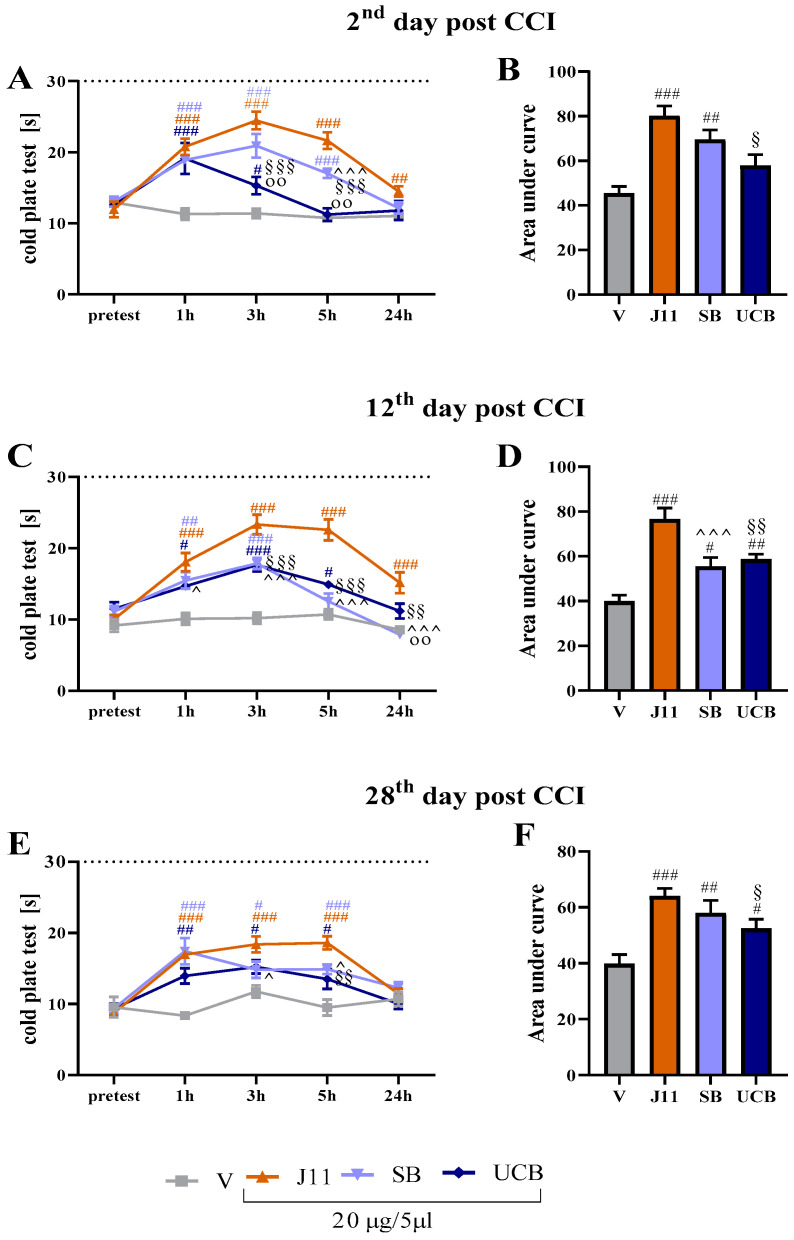
Effect of a single (20 μg/5 μL) *i.t*. administration of J113863, SB328437 and UCB35625 on thermal hypersensitivity (**A**,**C**,**E**) in male mice as measured on the 2nd, 12th, and 28th days after chronic constriction injury of the sciatic nerve. Measurements were performed 1, 3, 5 and 24 h after substance injection. The horizontal dotted line shows the cutoff value. Moreover, the data obtained were analyzed as areas under the curve (**B**,**D**,**F**). The data are presented as the means ± SEMs (**A**,**C**,**E**) or total area under curve ± SEMs (**B**,**D**,**F**). Intergroup differences were analyzed using one-way ANOVA with Bonferroni’s post hoc test for multiple comparisons. # *p* < 0.05, ## *p* < 0.01, and ### *p* < 0.001 indicate a significant difference compared with the vehicle-treated group. ^ *p* < 0.05, and ^^^ *p* < 0.001 indicate differences between J11 and SB, § *p* < 0.05, §§ *p* < 0.01, and §§§ *p* < 0.001 indicate differences between J11 and UCB; ᴏᴏ *p* < 0.01 indicates a significant difference between SB and UCB. The quantity of animals used in the experiment was as follows: 2nd day: V (*n* = 10), J11 (*n* = 8), SB (*n* = 9–10), UCB (*n* = 6); 12th day: V (*n* = 7), J11 (*n* = 7), SB (*n* = 8–10), UCB (*n* = 5); 28th day: V (*n* = 6), J11 (*n* = 5), SB (*n* = 7), UCB (*n* = 5). Abbreviations: J11—J113863, SB—SB328437, UCB—UCB35625, V—vehicle.

As shown in Figure 1, sciatic nerve surgery led to the development of mechanical and thermal hypersensitivity in mice, which was observed between the 2nd and 28th day. On the 2nd day after surgery, J113863, SB328437 and UCB35625 exerted analgesic effects compared to vehicle-treated animals as measured by von Frey (Figure 7A) and cold plate (Figure 8A) tests. However, in the case of mechanical hypersensitivity, the strongest effects were obtained for SB328437 (F = 5.69, *p* = 0.012, Figure 7B), and the peak of its action was observed 3 h (F = 14.30, *p* < 0.0001, Figure 7A) after injection, while in the case of thermal hypersensitivity, the highest effectiveness was observed for J11328437 (F = 12.02, *p* = 0.0006, Figure 8B), with the strongest effect measured 3 h (F= 37.92, *p* < 0.0001, Figure 8A) after substance delivery. Moreover, on the 12th day post CCI, all the tested substances showed antinociceptive properties according to the von Frey (Figure 7C) and cold plate (Figure 8C) test results. 

However, in both cases, J113863 showed the strongest analgesic effect (F = 7.44, *p* = 0.0045, Figure 7D and F = 16.89, *p* = 0.0001, Figure 8D). In the von Frey test, J113863 had a peak of action 5 h after its delivery (F = 10.09, *p* = 0.0002, Figure 7C), while in the cold plate test, the peak was observed after 3 h (F = 29.85, *p* < 0.0001, Figure 8C). On the 28th day after sciatic nerve surgery, J113863 and SB328437 had analgesic effects on mechanical stimulation, while less activity was observed for UCB35625 (Figure 7E). Between these two, SB328437 seems to exert the strongest effect (F = 4.19, *p* = 0.031, Figure 7F), and the highest effectiveness was obtained 5 h after injection (F = 7.00, *p* = 0.0023, Figure 7E). In the cold plate test, the most effective substance was J11328437 (F = 8.59, *p* = 0.0026, Figure 8F), with a peak of action 5 h after its delivery (F = 13.02, *p* < 0.0001, Figure 8E). 

In contrast to the von Frey test, UCB35625 showed some analgesic effects against thermal stimuli. Moreover, two-way ANOVA confirmed a significant interaction between the treatment and the analyzed time points in the von Frey test on the day 2nd, 12th and 28th (F = 7.58, *p* < 0.0001; F = 5.74, *p* < 0.0001; F = 3.68, *p* = 0.0001; respectively) and cold plate test at the same days (F = 8.64, *p* < 0.0001; F = 6.29, *p* < 0.0001; F = 4.17, *p* < 0.0001; respectively). Post hoc comparisons for this analysis are available in Supplementary S1.

Although, in the V-treated CCI-exposed group we observe impaired motor functions (Table 2) compared to naive animals (262.17 s ± 9.90—281.17 s ± 11.97, data not shown in the table). The single *i.t.* administration of J113863, SB328437 or UCB35625 did not influence the motor disfunction observed in V-treated CCI-exposed animals, as measured by the rotarod test on the 2nd, 12th and 28th days after chronic constriction injury of the sciatic nerve (Table 2).

### 3.8. Comparison of a Single i.t. J113863, SB328437, UCB35625 and J113863 + SB328437 Administration on % Maximal Possible Effect Measured 12 Days after Chronic Constriction Injury in Male and Female Mice

On the 12th day after surgery, J113863, SB328437, UCB35625 and J113863 + SB328437 exerted analgesic effects compared to vehicle-treated animals as measured by von Frey test in male mice (Table 3). In this case, the maximum possible range of antinociceptive effect was observed for J11 (F = 6.63, *p* = 0.0005). In the case of cold plate, J113863, SB328437 and UCB35625 revealed analgesic effects after its administration with the highest % MPE again observed for J113863 (F = 25.90, *p* < 0.0001). In the female animals, J113863, SB328437, and J113863 + SB328437 had analgesic effects compared with vehicle-treated animals as measured by von Frey test. The highest % MPE was obtained for SB328437 (F = 3.16, *p* = 0.029). Moreover, J113863, SB328437 and UCB35625 were effective in the case of cold plate test with the highest % MPE for SB328437 (F = 3.00 *p* = 0.035). Comparison of the % of the maximal possible effect between genders had revealed differences between effectiveness of J113863, showing its better effectiveness in male mice (t = 3.28, *p* = 0.0073). In the case of cold plate test, interaction between drug × sex was confirmed (F = 4.57, *p* = 0.0027). Post hoc comparisons for this analysis are available in Supplementary S1.

## 4. Discussion

Our research is the first comprehensive behavioral and biochemical study showing that many endogenous ligands of CCR1 (CCL2/3/5/7/8/9) and CCR3 (CCL5/7/8) are significant in the development (CCL2/3/5/7/8/9) and maintenance (CCL2/7/8) of neuropathic pain after peripheral nerve injury in mice. Among them, CCL2/7/8 seem to be the most important, because we detected a significant time-dependent and long-lasting increase in CCL2/7/8 protein levels from shortly after performing CCI to up to 28 days after surgery. Furthermore, intrathecal injections of these three chemokines evoked very strong hypersensitivity to mechanical and thermal stimuli. Additionally, for the first time, we report beneficial effects of bindarit, which inhibits the production of CCL2/7/8. Its analgesic properties were observed after single and, to a better degree, after repeated injections in the early and in the late phase of neuropathy development. Additionally, our results demonstrate the importance of CCR1 and CCR3 because their selective and dual antagonists were able to diminish mechanical and thermal hypersensitivity evoked by CCI in males and females. The obtained results provide evidence that CCR1, CCR3 and/or their ligands (CCL2/7/8) may serve as potential therapeutic targets for neuropathic pain treatment regardless of gender.

For several years, chemokines and their receptors have been gaining popularity in research on neuropathic pain of different etiologies. Investigations have been carried out in various animal models [26,30,38] and in patients [24,39], and this research direction seems to be very promising because a link between chemokines and pain can be clearly seen. However, to date, no drugs targeting the chemokine system are used clinically for the treatment of neuropathy, which in our opinion is related to an insufficient understanding of the roles and mechanisms of chemokine systems in pathological nociceptive processes.

**Scheme 6:**
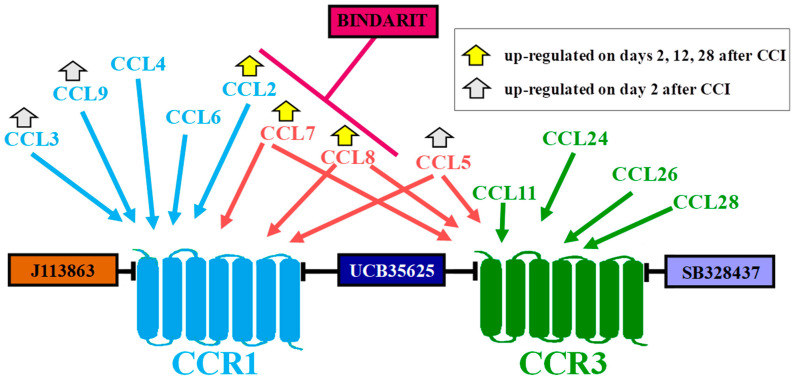
Selective inhibition of CCL2/7/8 production by bindarit, as well as blocking of CCR1 and CCR3 by selective (J113863, SB328437) or dual (UCB35625) antagonists, reduced even fully developed mechanical and thermal hypersensitivity in murine model of neuropathic pain.

First, our research provided evidence that the majority of CCR1 and CCR3 ligands are altered in the course of neuropathy in mice. In addition, for the first time, carrying out an analysis in one study enabled us to compare their pronociceptive effects. The results indicated that the protein with the greatest expression changes was CCL7, which acts by binding both CCR1 and CCR3 (Scheme 6).

Our results indicate that this chemokine participates in the initialization and maintenance of neuropathic pain after nerve ligation because enhanced CCL7 mRNA/protein levels were observed between the 2nd and 28th days of neuropathy. CCL7 may be released from macrophages, neurons and astrocytes [21,40,41] and can evoke chemotaxis or activation of other cells, e.g., microglia, macrophages and neutrophils [42,43,44], which are known to be important in neuropathy [3,45,46,47,48,49,50]. In our study, alterations in the number of these cells and/or their activation were observed, which may indicate that these cells are responsible for the secretion of CCL7 or might be attracted by it. Additionally, in our behavioral study, we showed the pronociceptive effects of this chemokine after a single *i.t.* injection, with hypersensitivity developing between 1 and 10 h after administration. Our study is in agreement with other research that has also suggested the pronociceptive properties of CCL7 [12]. In the spinal nerve ligation (SNL) model, increased spinal expression of CCL7 neurons has been demonstrated. CCL7 knockout mice developed neuropathic pain behavior to a lesser extent following SNL [21]. Importantly, from a clinical point of view, neutralization of CCL7 by its antibody was able to decrease pain-like behaviors in the model of neuropathy [12]. In our research, similar to CCL7, the chemokine CCL8, which binds both CCR1 and CCR3, is one of the most highly elevated cytokines in the spinal cord. Nevertheless, to the best of our knowledge, there are only a few studies on the role of CCL8 in painful neuropathy. The CCL8 level has already been reported to be increased in the cerebrospinal fluid of patients with neuropathic pain [24]. Thus far, our study is the only one that has shown that CCL8 injected intrathecally causes mechanical and thermal hypersensitivity with greater potency than other chemokines. In the case of thermal stimulation, pain-related behaviors were observed even 24 h after chemokine administration. The expression of this chemokine is observed in neurons and macrophages [25,51]. Based on these data, we suggest that CCL8 may be one of the most important chemokines in neuropathy development, especially because it is also known that inhibition of CCL8 may decrease visceral hyperalgesia [25]; however, more studies are needed to investigate its exact role in nociception. The third highly upregulated cytokine in our research acting via CCR1 was CCL2, a well-described chemokine involved in nociception. CCL2 may be secreted from various cells involved in neuropathic pain, such as microglia, macrophages, neutrophils, astrocytes and neurons [52,53,54,55]. Changes in the level of this chemokine in the spinal cord were observed between the 2nd and 28th days of neuropathy induced by sciatic nerve injury, which coincided with changes in IBA-1, GFAP and MPO levels. Importantly, the role of CCL2 in the central sensitization process, which causes neuropathic pain, has already been described [56]. Additionally, this chemokine is one of the main factors activating microglia and thus contributing to nociception [57]. Our research indicates that administration of CCL2 to naive animals evokes long-lasting pain-like symptoms even up to 24 h postinjection. Its pronociceptive properties have already been suggested by others, who reported that neutralization of CCL2 decreases pain-like behaviors [12]. Upregulation of this chemokine was also observed in neuropathy evoked by diabetes and chemotherapy [35,58]. Our current pharmacological studies have revealed that, among other chemokines, CCL2, CCL7 and CCL8 induce strong pain-related behaviors in naive mice; thus, we examined the pharmacological effect of a substance that selectively inhibits the production of these three chemokines, namely, bindarit (Scheme 6). This substance has been shown to have anti-inflammatory activity in the treatment of several experimental inflammatory and autoimmune disorders, from viral and adjuvant arthritis to acute pancreatitis [59,60,61]. Currently, bindarit is in the second phase of clinical trials for type 2 diabetic nephropathy patients (NCT01109212). In 2018, Liu et al. showed that bindarit suppresses cancer development and diminished pain in mice [62]. Bindarit was further effective in attenuating clinical experimental autoimmune encephalomyelitis in mice by suppressing the elevation of CCL2 in the brain and spinal cord [63]. Importantly, our data show that repeated injections of bindarit effectively attenuated pain-related behaviors in different phases of neuropathic pain development in mice. It brought analgesic effects even in fully developed neuropathy. However, a single injection of bindarit was not as effective. We observed that the highest dose of the tested substance decreased thermal to a greater extent than mechanical hypersensitivity, which may depend on different factors. First, the feeling of pain sensations depends on the targeting of different nerve fibers [64]. Our results suggest that bindarit may have a greater impact on Aδ and C fibers, which are responsible for cold sensation [65], with less effect on Aβ fibers, which are responsible for pathological touch feeling [66]. Furthermore, there is evidence that various cytokines may stimulate nerve fibers, leading to activation of the pain pathway [67,68]. Thus, it seems that inhibition of CCL2/7/8 synthesis might not alleviate mechanical hypersensitivity to the extent that it alleviates thermal. Nevertheless, the exact mechanism underlying bindarit activity remains unexplored, and further experiments are needed. A previous study identified a modulatory effect of bindarit on the nuclear factor-κB (NF-κB) signaling pathway in a macrophage cell line, specifically by inhibiting p65 and the p65/p50-mediated CCL2 promoter [69]. Additionally, Iwasawa et al. demonstrated that bindarit, by blocking NF-κB activation, suppresses microglial activation via downstream pronociceptive cytokines [70], which may consequently reduce neuropathic pain behavior [71,72]. Moreover, in the CCI model, previous work confirmed that NF-κB participates in the development of neuropathic pain [73,74] and that its inhibitors evoke pain relief [75,76]. This may be a possible explanation for the analgesic effect we observed in our study. Although the nerve damage caused long-term motor weakness, bindarit did not disturb motor coordination. Thus, the observed results indicate its analgesic properties. Our data are consistent with the widely proposed critical role of CCL2/7/8 in neuroinflammation and suggest that bindarit might have a therapeutic success in the treatment of immunological disorders.

Other chemokines belonging to the macrophage inflammatory protein (MIP) subfamily, namely, CCL3, 4 and 9, all of which act through CCR1, were also found to be altered, although not as strongly as CCL2/7/8. CCL3 is secreted from numerous cell types, e.g., microglia, neurons, neutrophils and T lymphocytes [55,77,78,79]. Our data showed upregulation of the mRNA expression of this chemokine from the 2nd until the 28th day; however, its protein level was only changed on the 2nd day, suggesting the importance of CCL3 in the initial phase of neuropathy, which correlates well with studies by Kiguchi et al. [80]. Notably, neutralization of this chemokine reduced pain-like behaviors in mice with diabetic neuropathy, which may indicate its role in this pathology [26]. Studies conducted in streptozotocin- and chemotherapy-induced neuropathy models have shown the participation of CCL4 in nociception [26,81]; however, in contrast, its protein levels do not increase in CCI-evoked neuropathy. Moreover, similar to CCL3, CCL9 seems to play an important role in the initial phase of neuropathy. Its contribution has been also confirmed in diabetic neuropathy [26]. Although CCL9 is expressed in rodents, the chemokine has a human ortholog, CCL23, whose upregulation was observed in the cerebrospinal fluid of patients with neuropathic pain [24]. Moreover, our data show that intrathecal injection of CCL9 may evoke mechanical/thermal hypersensitivity in naive mice. Our results indicate that among CCR1 ligands from the MIP family, CCL3 and CCL9 play an important role in the initiation of neuropathy, in addition to the previously mentioned CCL2/7/8, which are responsible for both the development and maintenance of pain.

The next examined cytokine was CCL5, an endogenous ligand for both CCR1 and CCR3. To date, upregulation of the CCL5 protein level after CCI has been shown in rats [22]; similarly, our studies in mice revealed significant alterations on the 2nd day post surgery. Moreover, it is known that CCL5 knockout mice develop lower hypersensitivity after partial sciatic nerve ligation [82], and CCL5 neutralization antibodies reduce pain-like behaviors [22,83]. In our studies, we demonstrated that CCL5 injection induced strong mechanical and even stronger thermal hypersensitivity, which lasted until 48 h after chemokine administration. Nevertheless, relatively smaller spinal changes in the CCL5 level suggest that its contribution to the pronociceptive effects mediated through CCR1 and CCR3 is less important than that of other ligands (especially CCL2/7/8).

Another CCR1 ligand that we studied is CCL6, whose spinal upregulation at the mRNA level was observed in all studied time points after nerve injury, similar to results obtained in rats [4,84]. However, we noticed little spinal downregulation of the CCL6 protein level on the 12th day, which may indicate a less important role of this chemokine in nociception. CCL6 is not present in humans, and similar to CCL9, it is considered as an ortholog of human CCL23; thus, it is worth examining CCL6 in other animal models of neuropathic pain to confirm or exclude its role.

We also investigated chemokines in the eotaxin family, namely, CCL11, CCL24, CL26, and CCL28, which all act via CCR3; however, we did not observe any alterations after nerve ligation. Nevertheless, others have shown that the level of CCL11 in synovial fluid is positively related to the pain score in osteoarthrosis [85]; moreover, the serum level of CCL24 was found to be higher in patients with fibromyalgia [86], which may suggest an important role of some eotaxins in different diseases accompanied by peripheral painful sensations. This action seems to be confirmed by the recently obtained results in rats, indicating that the expression of CCL11 does not change at the spinal cord level, but increases in the DRG [3]. The obtained results are important because they indicate that CCR3 ligands in the eotaxin family are not good targets for neuropathic pain relief.

As our studies demonstrated, a significant proportion of CCR1 and CCR3 ligands are upregulated during neuropathic pain development, which suggests the importance of these receptors in the pathogenesis of neuropathy. It was previously shown that these receptors are expressed by cells significant in nociceptive processes, such as microglia, macrophages, astrocytes, neurons and neutrophils [26,87,88,89,90,91,92,93]. Moreover, some studies have demonstrated that targeting chemokine receptors may positively impact neuropathic pain pathogenesis. Thus far, it has been shown that blockade of CCR2, CCR4, and CCR5 by antagonists diminished pain-like behaviors and simultaneously influenced the level of factors with pronociceptive properties and the number of activated cells [6,13,14,15]. Our results did not show upregulation of CCR1 and CCR3 between the 2nd and 28th days post CCI. Nevertheless, we observed analgesic effects after targeting both receptors. One explanation for the observed effects may be the internalization of receptors [94]. Despite the lack of changes in the protein level in the whole homogenate, the internalization process cannot be ruled out. We hypothesize that in both cases, the majority of receptors are on the cell surface; therefore, we observed strong analgesic effects of the tested antagonists. However, further studies are required to confirm our hypothesis. Previously, we indicated that single (12th day post CCI) and repeated (for 3 and 8 days) *i.t.* injection of CCR1 (J113863) [4] and CCCR3 (SB328437) [3] antagonists in rats beneficially impact neuropathic pain symptoms. In our current study, we showed that a single administration of these substances diminished mechanical and thermal hypersensitivity in mice in the early (2nd day post CCI) and late phase, when neuropathy is fully developed (28th day post CCI), which seems to be a substantial advantage of the examined substances. Additionally, CCR1 antagonists may have decreased pain-like behaviors in a murine model of diabetic neuropathy [26]. Currently, with the exception of our studies, there are no data on the role of CCR3 in other neuropathic pain models. J113863 and SB328437 did not disturb motor coordination in animals, which is undoubtedly a large advantage.

We were also encouraged by others to check whether blockade of more than one chemokine receptor at the same time may bring better analgesic effects than blocking receptors separately. Cenicriviroc, a dual antagonist of CCR2 and CCR5, was the most effective substance in attenuating painful sensations after a single injection compared to selective CCR2 (RS504393) and CCR5 (maraviroc) antagonists [13]. In the present study, we used a dual CCR1 and CCR3 antagonist, namely, UCB35625. To the best of our knowledge, this compound has never before been tested in any pain model. We observed for the first time that UCB35625 (CCR1/CCR3 antagonist) inhibits pain-like behavior, without influencing the motor dysfunction observed in neuropathy, which confirms its analgesic effectiveness. However, both UCB35625 as well as coadministration of J113863 and SB328437 (but only regarding the cold plate test) are surprisingly less effective than the selective antagonists J113863 and SB328437 administered separately, despite using the same doses. This is a very intriguing observation, which requires further in-depth investigations. We can propose a few hypotheses explaining the different effects of these tested substances. First, the effect may depend on the strength of the substance binding to the receptor, which may evoke different stronger or weaker pharmacological effects [95,96], and may also depend on the efficacy, which reflects the capacity of a substance to activate a receptor and generate a cellular response [97]. In the case of coadministration of substances, we cannot completely rule out the interaction of antagonists with each other, which is why the pharmacological effects may differ, while compared to separate administrations. Additionally, it is important to know the chemical structure of substances and their receptors since it can impact the obtained pharmacological response [96]. 

## 5. Conclusions

Overall, a deeper understanding of the role of chemokines and their temporal fluctuations is pivotal for the development of novel therapeutic strategies for neuropathy. Here, we presented significant evidence of the important role of the studied chemokines in the development (CCL2/3/5/7/8/9) and persistence (CCL2/7/8) of neuropathic pain. Moreover, selective inhibition of CCL2/7/8 production by bindarit, as well as blocking of CCR1 and CCR3 by selective (J113863, SB328437) or dual (UCB35625) antagonists, reduced even fully developed mechanical and thermal hypersensitivity, both after repeated and single administration. In light of our research, we propose that CCL2/7/8/CCR1 and CCL7/8/CCR3 signaling are important elements in the modulation of neuropathic pain. The results indicate that these chemokines and their receptors may be promising targets in the search for pharmacotherapeutic agents to treat neuropathic pain.

## Figures and Tables

**Table 1:** Effects of single (10, 20 and 40 μg/5 μL) and repeated (10 and 40 μg/5 μL) *i.t.* administration of bindarit on motor coordination on the 2nd, 12th and 28th days after CCI of the sciatic nerve in male mice. Measurements in a rotarod apparatus were performed 2 h after single drug administration or 2 h after three administrations of the substance. The data are presented as the means ± SEMs. Intergroup differences were analyzed using one-way ANOVA with Bonferroni’s post hoc test for multiple comparisons. ^#^ *p* < 0.05 indicates a significant difference compared with the vehicle-treated group. The quantity of animals used in the experiment was as follows: Single injection: (2nd day: V (*n* = 6), B10 (*n* = 6), B20 (*n* = 6), B40 (*n* = 6); 12th day: V (*n* = 7), B10 (*n* = 6), B20 (*n* = 7), B40 (*n* = 6), 28th day: V (*n* = 6), B10 (*n* = 6), B20 (*n* = 6), B40 (*n* = 7)); Repeated injection: (2nd day: V (*n* = 10), B10 (*n* = 10), B40 (*n* = 10); 12th day: V (*n* = 9), B10 (*n* = 10), B40 (*n* = 8); 28th day: V (*n* = 5), B10 (*n* = 6), B40 (*n* = 6)). Abbreviations: B—Bindarit, V—vehicle.

	Substance	Bindarit—Single Injection Rotarod Test (s)				Bindarit—Repeated Injection Rotarod Test (s)		
Day		V	B10	B20	B40	V	B10	B40
2nd		162.33 ± 27.83	149.67 ± 11.54	229.67 ± 18.76 ^#^	163.14 ± 19.20	175.00 ± 24.19	186.50 ± 18.867	186.60 ± 19.52
12th		98.57 ± 20.62	138.83 ± 28.74	78.00 ± 14.64	133.58 ± 37.75	141.00 ± 20.14	133.40 ± 18.05	176.36 ± 21.41
28th		102.83 ± 27.39	114.42 ± 24.58	120.67 ± 15.95	100.14 ± 27.78	97.2 ± 27.39	190.83 ± 27.27 ^#^	134.33 ± 18.15

**Table 2:** Effect of a single (20 μg/5 μL) *i.t.* administration of J113863, SB328437 or UCB35625 on motor coordination in male mice on the 2nd, 12th and 28th days after chronic constriction injury of the sciatic nerve. Measurements in a rotarod apparatus were performed 2 h after single drug administration. The data are presented as the means ± SEMs. Intergroup differences were analyzed using one-way ANOVA with Bonferroni’s post hoc test for multiple comparisons. The quantity of animals used in the experiment was as follows: 2nd day: V (*n* = 10), J11 (*n* = 7), SB (*n* = 10), UCB (*n* = 6); 12th day: V (*n* = 7), J11 (*n* = 8), SB (*n* = 10), UCB (*n* = 5); 28th day: V (*n* = 6), J11 (*n* = 5), SB (*n* = 7), UCB (*n* = 5). Abbreviations: J11—J113863, SB—SB328437, UCB—UCB35625, V—vehicle.

	Substance	J113863, SB328437, UCB35625—Single Injection Rotarod Test (s)			
Day		V	J11	SB	UCB
2nd		153.00 ± 18.38	183.00 ± 26.28	161.06 ± 24.17	133.33 ± 30.96
12th		185.29 ± 15.59	171.14 ± 25.42	155.20 ± 16.27	137.80 ± 23.89
28th		164.17 ± 20.63	110.30 ± 17.57	167.00 ± 19.78	170.20 ± 24.53

**Table 3:** Effect of a single (20 μg/5 μL) *i.t.* administration of J113863, SB328437, UCB35625 or J11386 + SB328437 (20 μg/5 μL + 20 μg/5) on % of the maximal possible effect on the 12th day after chronic constriction injury of the sciatic nerve in male and female mice. Measurements in a von Frey and cold plate apparatus were performed 3 h after single drug administration. The data are presented as the means ± SEMs. Intergroup differences were analyzed using one-way ANOVA with Bonferroni’s post hoc test for multiple comparisons. *t* test was used to evaluate sex differences in each substance. ^#^ *p* < 0.05, ^##^ *p* < 0.01 and ^###^ *p* < 0.001 indicate a significant difference compared with the vehicle group; ^§§^ *p* < 0.01 indicates a significant difference between male and female. The quantity of animals used in the experiment was as follows: male mice: (von Frey: V (*n* = 10), J11 (*n* = 7), SB (*n* = 10), UCB (*n* = 5), J11 + SB (*n* = 8)), (cold plate: V (*n* = 7), J11 (*n* = 8), SB (*n* = 8), UCB (*n* = 5); J11 + SB (*n* = 8)); female mice: (von Frey: V (*n* = 8), J11 (*n* = 6), SB (*n* = 8), UCB (*n* = 6), J11 + SB (*n* = 6), (cold plate: V (*n* = 8), J11 (*n* = 6), SB (*n* = 8), UCB (*n* = 6); J11 + SB (*n* = 6)). Abbreviations: J11—J113863, SB—SB328437, UCB—UCB35625, V—vehicle.

	Substances	% of the Maximal Possible Effect				
Sex	Test	V	J11	SB	UCB	J11 + SB
**♂**	von Frey	−0.5 ± 0.2	5.9 ± 1.2 ^###^	2.4 ± 0.5 ^#^	4.5 ± 1.1 ^##^	4.3 ± 1.7 ^###^
	cold plate	2.1 ± 4.5	65.5 ± 7.5 ^###^	36.2 ± 3.7 ^###^	33.1 ± 4.9 ^###^	11.1 ± 3.7
**♀**	von Frey	0.2 ± 0.6	5.7 ± 1.3 ^#^	6.2 ± 2.0 ^##^	3.4 ± 1.3	5.0 ± 1.3 ^#^
	cold plate	2.1 ± 4.5	24.5 ± 10.3 ^# §§^	32.6 ± 7.8 ^##^	24.5 ± 3.8 ^#^	19.0 ± 7.6

## Data Availability

The data presented in this study are available on request from the corresponding author.

## Supplementary Materials

The following supporting information can be downloaded at: https://www.mdpi.com/article/10.3390/cells12010098/s1, S1: Two-way Analysis of Variance for Tables/Figures according to post-hoc test.

## Abbreviations

ANOVA	Analysis of variance
AUC	Area under the curve
CCI	Model of chronic constriction injury to the sciatic nerve
CCL	CC motif chemokine ligand
CCR	CC chemokine receptor type
CD	Cluster of differentiation
cDNA	Complementary deoxyribonucleic acid
DMSO	Dimethyl sulfoxide
DRG	Dorsal root ganglia
ELISA	Enzyme-linked immunosorbent assay
GAPDH	Glyceraldehyde 3-phosphate dehydrogenase
GFAP	Glial fibrillary acidic protein
HPRT	Hypoxanthine phosphoribosyltransferase
IBA-1	Ionized calcium-binding adapter molecule-1
*i.t.*	Intrathecal drug administration
L4-L6	Lumbar 4–6 part of the spinal cord
MIP	Macrophage inflammatory protein
MPO	Myeloperoxidase
mRNA	messenger ribonucleic acid
N	Naive
NF-κB	Nuclear factor kappa light-chain-enhancer of activated B cells
RT-qPCR	Quantitative reverse transcription polymerase chain reaction
SEM	Standard error of the mean
SNL	Spinal nerve ligation
TBST	Tris-buffered saline with tween
V	Vehicle
