# Supplementary material for: Pharmacological Evidence of the Important Roles of CCR1 and CCR3 and Their Endogenous Ligands CCL2/7/8 in Hypersensitivity Based on a Murine Model of Neuropathic Pain
Source: Cells. 2022 Dec 26;12(1):98. doi: 10.3390/cells12010098 (PMC9818689; doi:10.3390/cells12010098)
Supplement: Supplementary file 1 [file cells-12-00098-s001.zip › cells-2021846-Supplementary S1.pdf]

1) Two-way Analysis of Variance for **Figure 4.A** according to *post -hoc* test

ANOVA results | **Multiple comparisons**

	2way ANOVA Multiple comparisons					
4	Number of comparisons per family	21				
5	Alpha	0.05				
6						
7	**Bonferroni's multiple comparisons test**	**Mean Diff.**	**95.00% CI of diff.**	**Below threshold?**	**Summary**	**Adjusted P Value**
8						
9	V vs. CCL2	2.427	1.954 to 2.901	Yes	****	<0.0001
10	V vs. CCL3	1.466	0.9923 to 1.940	Yes	****	<0.0001
11	V vs. CCL5	1.356	0.8822 to 1.830	Yes	****	<0.0001
12	V vs. CCL7	2.110	1.636 to 2.583	Yes	****	<0.0001
13	V vs. CCL8	2.276	1.802 to 2.750	Yes	****	<0.0001
14	V vs. CCL9	1.258	0.7846 to 1.732	Yes	****	<0.0001
15	CCL2 vs. CCL3	-0.9613	-1.435 to -0.4876	Yes	****	<0.0001
16	CCL2 vs. CCL5	-1.071	-1.545 to -0.5977	Yes	****	<0.0001
17	CCL2 vs. CCL7	-0.3179	-0.7916 to 0.1559	No	ns	0.8561
18	CCL2 vs. CCL8	-0.1515	-0.6252 to 0.3223	No	ns	>0.9999
19	CCL2 vs. CCL9	-1.169	-1.643 to -0.6953	Yes	****	<0.0001
20	CCL3 vs. CCL5	-0.1101	-0.5839 to 0.3636	No	ns	>0.9999
21	CCL3 vs. CCL7	0.6435	0.1697 to 1.117	Yes	***	0.0009
22	CCL3 vs. CCL8	0.8098	0.3361 to 1.284	Yes	****	<0.0001
23	CCL3 vs. CCL9	-0.2077	-0.6815 to 0.2660	No	ns	>0.9999
24	CCL5 vs. CCL7	0.7536	0.2798 to 1.227	Yes	****	<0.0001
25	CCL5 vs. CCL8	0.9199	0.4462 to 1.394	Yes	****	<0.0001
26	CCL5 vs. CCL9	-0.09762	-0.5714 to 0.3761	No	ns	>0.9999
27	CCL7 vs. CCL8	0.1664	-0.3074 to 0.6401	No	ns	>0.9999
28	CCL7 vs. CCL9	-0.8512	-1.325 to -0.3774	Yes	****	<0.0001
29	CCL8 vs. CCL9	-1.018	-1.491 to -0.5438	Yes	****	<0.0001

2) Two-way Analysis of Variance for **Figure 4.B** according to *post -hoc* test

	2way ANOVA Multiple comparisons					
4	Number of comparisons per family	21				
5	Alpha	0.05				
6						
7	**Bonferroni's multiple comparisons**	**Predicted (LS) mean diff.**	**95.00% CI of diff.**	**Below threshold?**	**Summary**	**Adjusted P Value**
8						
9	V vs. CCL2	6.213	4.553 to 7.873	Yes	****	<0.0001
10	V vs. CCL3	6.699	5.040 to 8.359	Yes	****	<0.0001
11	V vs. CCL5	6.606	4.938 to 8.274	Yes	****	<0.0001
12	V vs. CCL7	6.803	5.143 to 8.463	Yes	****	<0.0001
13	V vs. CCL8	8.658	6.999 to 10.32	Yes	****	<0.0001
14	V vs. CCL9	5.283	3.590 to 6.977	Yes	****	<0.0001
15	CCL2 vs. CCL3	0.4863	-1.173 to 2.146	No	ns	>0.9999
16	CCL2 vs. CCL5	0.3931	-1.275 to 2.061	No	ns	>0.9999
17	CCL2 vs. CCL7	0.5899	-1.070 to 2.250	No	ns	>0.9999
18	CCL2 vs. CCL8	2.445	0.7856 to 4.105	Yes	***	0.0002
19	CCL2 vs. CCL9	-0.9297	-2.623 to 0.7635	No	ns	>0.9999
20	CCL3 vs. CCL5	-0.09320	-1.761 to 1.575	No	ns	>0.9999
21	CCL3 vs. CCL7	0.1036	-1.556 to 1.763	No	ns	>0.9999
22	CCL3 vs. CCL8	1.959	0.2993 to 3.619	Yes	**	0.0073
23	CCL3 vs. CCL9	-1.416	-3.109 to 0.2772	No	ns	0.2289
24	CCL5 vs. CCL7	0.1968	-1.471 to 1.865	No	ns	>0.9999
25	CCL5 vs. CCL8	2.052	0.3840 to 3.720	Yes	**	0.0041
26	CCL5 vs. CCL9	-1.323	-3.024 to 0.3787	No	ns	0.3753
27	CCL7 vs. CCL8	1.855	0.1957 to 3.515	Yes	*	0.0146
28	CCL7 vs. CCL9	-1.520	-3.213 to 0.1736	No	ns	0.1329
29	CCL8 vs. CCL9	-3.375	-5.068 to -1.682	Yes	****	<0.0001

3) Two-way Analysis of Variance for **Figure 5.A** according to *post -hoc* test

	2way ANOVA<br>Multiple comparisons					
4	Number of comparisons per family	6				
5	Alpha	0.05				
6						
7	**Bonferroni's multiple comparisons test**	**Predicted (LS) mean**	**95.00% CI of diff.**	**Below threshold?**	**Summary**	**Adjusted P Value**
8						
9	V vs. B10	-0.1278	-0.2831 to 0.02759	No	ns	0.1755
10	V vs. B20	-0.1444	-0.3036 to 0.01476	No	ns	0.0984
11	V vs. B40	-0.2583	-0.4137 to -0.1030	Yes	***	0.0001
12	B10 vs. B20	-0.01667	-0.1759 to 0.1425	No	ns	>0.9999
13	B10 vs. B40	-0.1306	-0.2859 to 0.02481	No	ns	0.1559
14	B20 vs. B40	-0.1139	-0.2731 to 0.04532	No	ns	0.3438
15						

4) Two-way Analysis of Variance for **Figure 5.B** according to *post -hoc* test

	2way ANOVA<br>Multiple comparisons					
4	Number of comparisons per family	3				
5	Alpha	0.05				
6						
7	**Bonferroni's multiple comparisons test**	**Mean Diff.**	**95.00% CI of diff.**	**Below threshold?**	**Summary**	**Adjusted P Value**
8						
9	V vs. B10	-0.5161	-0.7145 to -0.3177	Yes	****	<0.0001
10	V vs. B40	-0.6994	-0.8978 to -0.5011	Yes	****	<0.0001
11	B10 vs. B40	-0.1833	-0.3817 to 0.01505	No	ns	0.0802

5) Two-way Analysis of Variance for **Figure 5.C** according to *post -hoc* test

	2way ANOVA<br>Multiple comparisons					
4	Number of comparisons per family	6				
5	Alpha	0.05				
6						
7	**Bonferroni's multiple comparisons test**	**Mean Diff.**	**95.00% CI of diff.**	**Below threshold?**	**Summary**	**Adjusted P Value**
8						
9	V vs. B10	-0.1095	-0.3084 to 0.08938	No	ns	0.8575
10	V vs. B20	-0.3905	-0.5894 to -0.1916	Yes	****	<0.0001
11	V vs. B40	-0.2119	-0.4108 to -0.01300	Yes	*	0.0301
12	B10 vs. B20	-0.2810	-0.4799 to -0.08205	Yes	**	0.0014
13	B10 vs. B40	-0.1024	-0.3013 to 0.09652	No	ns	>0.9999
14	B20 vs. B40	0.1786	-0.02033 to 0.3775	No	ns	0.1056

6) Two-way Analysis of Variance for **Figure 5.D** according to *post -hoc* test

	2way ANOVA<br>Multiple comparisons					
4	Number of comparisons per family	3				
5	Alpha	0.05				
6						
7	**Bonferroni's multiple comparisons test**	**Predicted (LS) mean diff.**	**95.00% CI of diff.**	**Below threshold?**	**Summary**	**Adjusted P Value**
8						
9	V vs. B10	-1.167	-1.543 to -0.7906	Yes	****	<0.0001
10	V vs. B40	-1.713	-2.110 to -1.315	Yes	****	<0.0001
11	B10 vs. B40	-0.5458	-0.9322 to -0.1595	Yes	**	0.0024

7) Two-way Analysis of Variance for **Figure 5.E** according to *post -hoc* test

	2way ANOVA Multiple comparisons					
4	Number of comparisons per family	6				
5	Alpha	0.05				
6						
7	**Bonferroni's multiple comparisons test**	**Predicted (LS) mean diff.**	**95.00% CI of diff.**	**Below threshold?**	**Summary**	**Adjusted P Value**
8						
9	V vs. B10	-0.01984	-0.2185 to 0.1788	No	ns	>0.9999
10	V vs. B20	-0.2810	-0.4796 to -0.08228	Yes	**	0.0014
11	V vs. B40	-0.1190	-0.3099 to 0.07183	No	ns	0.5830
12	B10 vs. B20	-0.2611	-0.4673 to -0.05494	Yes	**	0.0055
13	B10 vs. B40	-0.09921	-0.2979 to 0.09946	No	ns	>0.9999
14	B20 vs. B40	0.1619	-0.03677 to 0.3606	No	ns	0.1849

8) Two-way Analysis of Variance for **Figure 5.F** according to *post -hoc* test

	2way ANOVA Multiple comparisons					
4	Number of comparisons per family	3				
5	Alpha	0.05				
6						
7	**Bonferroni's multiple comparisons test**	**Predicted (LS) mean diff.**	**95.00% CI of diff.**	**Below threshold?**	**Summary**	**Adjusted P Value**
8						
9	V vs. B10	-1.217	-1.822 to -0.6112	Yes	****	<0.0001
10	V vs. B40	-2.689	-3.294 to -2.083	Yes	****	<0.0001
11	B10 vs. B40	-1.472	-2.049 to -0.8949	Yes	****	<0.0001
12						

9) Two-way Analysis of Variance for **Figure 6.A** according to *post -hoc* test

	2way ANOVA Multiple comparisons					
4	Number of comparisons per family	6				
5	Alpha	0.05				
6						
7	**Bonferroni's multiple comparisons test**	**Predicted (LS) mean diff.**	**95.00% CI of diff.**	**Below threshold?**	**Summary**	**Adjusted P Value**
8						
9	V vs. B10	-3.363	-5.238 to -1.487	Yes	****	<0.0001
10	V vs. B20	-4.165	-6.087 to -2.243	Yes	****	<0.0001
11	V vs. B40	-5.885	-7.760 to -4.009	Yes	****	<0.0001
12	B10 vs. B20	-0.8022	-2.724 to 1.120	No	ns	>0.9999
13	B10 vs. B40	-2.522	-4.398 to -0.6467	Yes	**	0.0027
14	B20 vs. B40	-1.720	-3.642 to 0.2018	No	ns	0.1073

10) Two-way Analysis of Variance for **Figure 6.B** according to *post -hoc* test

	2way ANOVA Multiple comparisons					
4	Number of comparisons per family	3				
5	Alpha	0.05				
6						
7	**Bonferroni's multiple comparisons test**	**Predicted (LS) mean diff.**	**95.00% CI of diff.**	**Below threshold?**	**Summary**	**Adjusted P Value**
8						
9	V vs. B10	-3.739	-4.955 to -2.523	Yes	****	<0.0001
10	V vs. B40	-7.609	-8.831 to -6.387	Yes	****	<0.0001
11	B10 vs. B40	-3.870	-5.092 to -2.648	Yes	****	<0.0001

11) Two-way Analysis of Variance for **Figure 6.C** according to *post -hoc* test

	2way ANOVA Multiple comparisons					
4	Number of comparisons per family	6				
5	Alpha	0.05				
6						
7	**Bonferroni's multiple comparisons test**	**Predicted (LS) mean diff.**	**95.00% CI of diff.**	**Below threshold?**	**Summary**	**Adjusted P Value**
8						
9	V vs. B10	-5.655	-7.729 to -3.581	Yes	****	<0.0001
10	V vs. B20	-6.386	-8.460 to -4.312	Yes	****	<0.0001
11	V vs. B40	-3.506	-5.594 to -1.418	Yes	****	<0.0001
12	B10 vs. B20	-0.7310	-2.805 to 1.343	No	ns	>0.9999
13	B10 vs. B40	2.149	0.06052 to 4.237	Yes	*	0.0400
14	B20 vs. B40	2.880	0.7915 to 4.968	Yes	**	0.0019

12) Two-way Analysis of Variance for **Figure 6.D** according to *post -hoc* test

	2way ANOVA Multiple comparisons					
4	Number of comparisons per family	3				
5	Alpha	0.05				
6						
7	**Bonferroni's multiple comparisons test**	**Predicted (LS) mean diff.**	**95.00% CI of diff.**	**Below threshold?**	**Summary**	**Adjusted P Value**
8						
9	V vs. B10	-8.299	-9.786 to -6.812	Yes	****	<0.0001
10	V vs. B40	-5.975	-7.546 to -4.403	Yes	****	<0.0001
11	B10 vs. B40	2.325	0.7981 to 3.851	Yes	***	0.0010
12						

13) Two-way Analysis of Variance for **Figure 6.E** according to *post -hoc* test

	2way ANOVA Multiple comparisons					
4	Number of comparisons per family	6				
5	Alpha	0.05				
6						
7	**Bonferroni's multiple comparisons test**	**Predicted (LS) mean diff.**	**95.00% CI of diff.**	**Below threshold?**	**Summary**	**Adjusted P Value**
8						
9	V vs. B10	-2.662	-4.191 to -1.132	Yes	****	<0.0001
10	V vs. B20	-3.314	-4.804 to -1.824	Yes	****	<0.0001
11	V vs. B40	-0.9786	-2.410 to 0.4529	No	ns	0.4158
12	B10 vs. B20	-0.6522	-2.237 to 0.9322	No	ns	>0.9999
13	B10 vs. B40	1.683	0.1535 to 3.213	Yes	*	0.0227
14	B20 vs. B40	2.335	0.8454 to 3.825	Yes	***	0.0003

14) Two-way Analysis of Variance for **Figure 6.F** according to *post -hoc* test

	2way ANOVA Multiple comparisons					
4	Number of comparisons per family	3				
5	Alpha	0.05				
6						
7	**Bonferroni's multiple comparisons test**	**Predicted (LS) mean diff.**	**95.00% CI of diff.**	**Below threshold?**	**Summary**	**Adjusted P Value**
8						
9	V vs. B10	-5.425	-7.736 to -3.114	Yes	****	<0.0001
10	V vs. B40	-7.961	-10.27 to -5.650	Yes	****	<0.0001
11	B10 vs. B40	-2.536	-4.739 to -0.3329	Yes	*	0.0184

15) Two-way Analysis of Variance for **Figure 7.A** according to *post -hoc* test

	2way ANOVA<br>Multiple comparisons					
4	Number of comparisons per family	6				
5	Alpha	0.05				
6						
7	**Bonferroni's multiple comparisons test**	**Predicted (LS) mean diff.**	**95.00% CI of diff.**	**Below threshold?**	**Summary**	**Adjusted P Value**
8						
9	V vs. J11	-0.7889	-1.152 to -0.4255	Yes	****	<0.0001
10	V vs. SB	-1.120	-1.460 to -0.7801	Yes	****	<0.0001
11	V vs. UCB	-0.7893	-1.182 to -0.3968	Yes	****	<0.0001
12	J11 vs. SB	-0.3311	-0.6945 to 0.03224	No	ns	0.0960
13	J11 vs. UCB	-0.0004760	-0.4135 to 0.4125	No	ns	>0.9999
14	SB vs. UCB	0.3307	-0.06183 to 0.7232	No	ns	0.1544

16) Two-way Analysis of Variance for **Figure 7.C** according to *post -hoc* test

	2way ANOVA<br>Multiple comparisons					
4	Number of comparisons per family	6				
5	Alpha	0.05				
6						
7	**Bonferroni's multiple comparisons test**	**Predicted (LS) mean diff.**	**95.00% CI of diff.**	**Below threshold?**	**Summary**	**Adjusted P Value**
8						
9	V vs. J11	-1.069	-1.412 to -0.7252	Yes	****	<0.0001
10	V vs. SB	-0.6820	-0.9986 to -0.3654	Yes	****	<0.0001
11	V vs. UCB	-0.5640	-0.9402 to -0.1878	Yes	***	0.0006
12	J11 vs. SB	0.3866	0.06999 to 0.7032	Yes	**	0.0082
13	J11 vs. UCB	0.5046	0.1284 to 0.8807	Yes	**	0.0028
14	SB vs. UCB	0.1180	-0.2339 to 0.4699	No	ns	>0.9999

17) Two-way Analysis of Variance for **Figure 7.E** according to *post -hoc* test

	2way ANOVA<br>Multiple comparisons					
4	Number of comparisons per family	6				
5	Alpha	0.05				
6						
7	**Bonferroni's multiple comparisons test**	**Predicted (LS) mean diff.**	**95.00% CI of diff.**	**Below threshold?**	**Summary**	**Adjusted P Value**
8						
9	V vs. J11	-0.4427	-0.7706 to -0.1147	Yes	**	0.0027
10	V vs. SB	-0.6181	-0.9194 to -0.3168	Yes	****	<0.0001
11	V vs. UCB	-0.2747	-0.6026 to 0.05331	No	ns	0.1579
12	J11 vs. SB	-0.1754	-0.4926 to 0.1417	No	ns	0.8364
13	J11 vs. UCB	0.1680	-0.1746 to 0.5106	No	ns	>0.9999
14	SB vs. UCB	0.3434	0.02628 to 0.6606	Yes	*	0.0264

18) Two-way Analysis of Variance for **Figure 8.A** according to *post -hoc* test

	2way ANOVA<br>Multiple comparisons					
4	Number of comparisons per family	6				
5	Alpha	0.05				
6						
7	**Bonferroni's multiple comparisons test**	**Predicted (LS) mean diff.**	**95.00% CI of diff.**	**Below threshold?**	**Summary**	**Adjusted P Value**
8						
9	V vs. J11	-7.169	-8.780 to -5.558	Yes	****	<0.0001
10	V vs. SB	-4.918	-6.446 to -3.391	Yes	****	<0.0001
11	V vs. UCB	-2.524	-4.278 to -0.7703	Yes	**	0.0011
12	J11 vs. SB	2.251	0.6318 to 3.869	Yes	**	0.0017
13	J11 vs. UCB	4.645	2.811 to 6.479	Yes	****	<0.0001
14	SB vs. UCB	2.394	0.6335 to 4.155	Yes	**	0.0023

19) Two-way Analysis of Variance for **Figure 8.C** according to *post -hoc* test

	2way ANOVA Multiple comparisons					
4	Number of comparisons per family	6				
5	Alpha	0.05				
6						
7	**Bonferroni's multiple comparisons test**	**Predicted (LS) mean diff.**	**95.00% CI of diff.**	**Below threshold?**	**Summary**	**Adjusted P Value**
8						
9	V vs. J11	-8.103	-9.783 to -6.423	Yes	****	<0.0001
10	V vs. SB	-3.237	-4.833 to -1.641	Yes	****	<0.0001
11	V vs. UCB	-4.240	-6.081 to -2.400	Yes	****	<0.0001
12	J11 vs. SB	4.866	3.270 to 6.462	Yes	****	<0.0001
13	J11 vs. UCB	3.863	2.022 to 5.703	Yes	****	<0.0001
14	SB vs. UCB	-1.003	-2.767 to 0.7611	No	ns	0.7787

20) Two-way Analysis of Variance for **Figure 8.E** according to *post -hoc* test

	2way ANOVA Multiple comparisons					
4	Number of comparisons per family	6				
5	Alpha	0.05				
6						
7	**Bonferroni's multiple comparisons test**	**Predicted (LS) mean diff.**	**95.00% CI of diff.**	**Below threshold?**	**Summary**	**Adjusted P Value**
8						
9	V vs. J11	-4.849	-6.669 to -3.029	Yes	****	<0.0001
10	V vs. SB	-3.775	-5.447 to -2.103	Yes	****	<0.0001
11	V vs. UCB	-2.437	-4.257 to -0.6166	Yes	**	0.0030
12	J11 vs. SB	1.074	-0.6861 to 2.834	No	ns	0.6206
13	J11 vs. UCB	2.412	0.5110 to 4.313	Yes	**	0.0056
14	SB vs. UCB	1.338	-0.4219 to 3.098	No	ns	0.2595

21) Two-way Analysis of Variance for **Tab. 3 (von Frey)** according to *post -hoc* test

	2way ANOVA Multiple comparisons					
4	Number of comparisons per family	5				
5	Alpha	0.05				
6						
7	**Bonferroni's multiple comparisons test**	**Predicted (LS) mean diff.**	**95.00% CI of diff.**	**Below threshold?**	**Summary**	**Adjusted P Value**
8						
9	male - female					
10	V	-0.8000	-4.980 to 3.380	No	ns	>0.9999
11	J11	0.2000	-4.702 to 5.102	No	ns	>0.9999
12	SB	-3.800	-8.206 to 0.6059	No	ns	0.1266
13	UCB	1.100	-4.236 to 6.436	No	ns	>0.9999
14	J11+SB	-0.7000	-5.459 to 4.059	No	ns	>0.9999

22) Two-way Analysis of Variance for **Tab. 3 (cold plate)** according to *post -hoc* test

	2way ANOVA Multiple comparisons					
4	Number of comparisons per family	5				
5	Alpha	0.05				
6						
7	**Bonferroni's multiple comparisons test**	**Predicted (LS) mean diff.**	**95.00% CI of diff.**	**Below threshold?**	**Summary**	**Adjusted P Value**
8						
9	male - female					
10	V	0.000	-20.31 to 20.31	No	ns	>0.9999
11	J11	41.00	17.18 to 64.82	Yes	***	0.0001
12	SB	3.600	-17.80 to 25.00	No	ns	>0.9999
13	UCB	8.600	-17.32 to 34.52	No	ns	>0.9999
14	J11+SB	-7.900	-31.02 to 15.22	No	ns	>0.9999
